# Overlapping group screening for detection of gene-environment interactions with application to TCGA high-dimensional survival genomic data

**DOI:** 10.1186/s12859-022-04750-7

**Published:** 2022-05-30

**Authors:** Jie-Huei Wang, Kang-Hsin Wang, Yi-Hau Chen

**Affiliations:** 1 Department of Statistics, Feng Chia University, Seatwen, Taichung, 40724 Taiwan; 2 Institute of Statistical Science, Academia Sinica, Nankang, Taipei, 11529 Taiwan

**Keywords:** Gene-environment interaction, Joint model, Lasso, Overlapping group screening, Survival prediction, TCGA

## Abstract

**Background:**

In the context of biomedical and epidemiological research, gene-environment (G-E) interaction is of great significance to the etiology and progression of many complex diseases. In high-dimensional genetic data, two general models, marginal and joint models, are proposed to identify important interaction factors. Most existing approaches for identifying G-E interactions are limited owing to the lack of robustness to outliers/contamination in response and predictor data. In particular, right-censored survival outcomes make the associated feature screening even challenging. In this article, we utilize the overlapping group screening (OGS) approach to select important G-E interactions related to clinical survival outcomes by incorporating the gene pathway information under a joint modeling framework.

**Results:**

Simulation studies under various scenarios are carried out to compare the performances of our proposed method with some commonly used methods. In the real data applications, we use our proposed method to identify G-E interactions related to the clinical survival outcomes of patients with head and neck squamous cell carcinoma, and esophageal carcinoma in The Cancer Genome Atlas clinical survival genetic data, and further establish corresponding survival prediction models. Both simulation and real data studies show that our method performs well and outperforms existing methods in the G-E interaction selection, effect estimation, and survival prediction accuracy.

**Conclusions:**

The OGS approach is useful for selecting important environmental factors, genes and G-E interactions in the ultra-high dimensional feature space. The prediction ability of OGS with the Lasso penalty is better than existing methods. The same idea of the OGS approach can apply to other outcome models, such as the proportional odds survival time model, the logistic regression model for binary outcomes, and the multinomial logistic regression model for multi-class outcomes.

**Supplementary Information:**

The online version contains supplementary material available at 10.1186/s12859-022-04750-7.

## Background

It is believed that in the development of complex diseases such as cancer, diabetes, and so on, gene-environment (G-E) interaction plays a critical role beyond the main genetic (G) or environmental (E) factors ([1, 2] and so on). For example, Batchelor et al. [3] showed that the interaction between the gene TP53 and age affects the prognosis of glioblastoma. As a consequence, incorporating significant G-E interaction factors into a survival prediction model would enhance the performance of the later.

In the setting of high-dimensional genetic data analysis, there exist two ways to identification of important G-E interactions: the marginal and joint analyses [4]. The marginal analysis considers only one gene at a time, and fits a model consisting of multiple E factors, this gene, and its interaction with E factors. the other performs joint analysis and considers all genes in a single model.

**Fig. 1:**
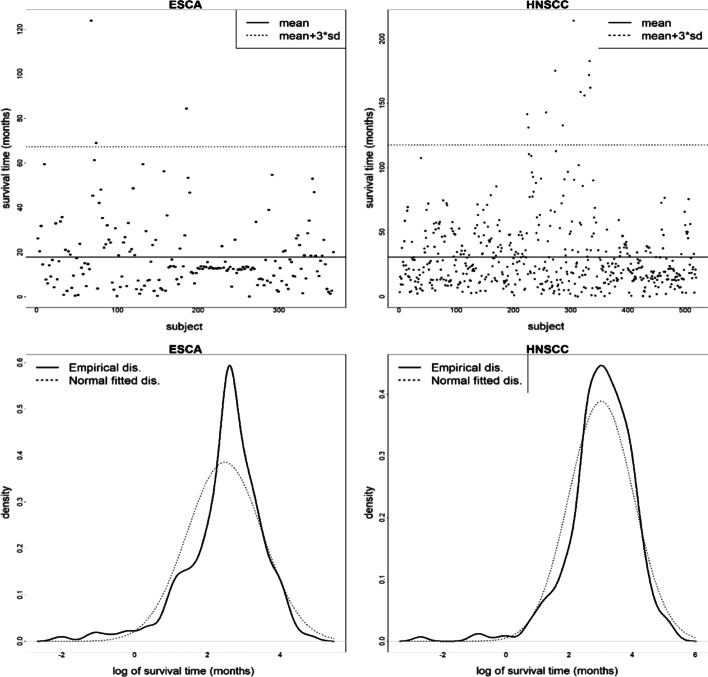
The long-tailed distribution of clinical survival data for the TCGA ESCA and HNSCC

In the framework of marginal analysis of high-dimensional genetic data, for each gene, a model consisting of multiple E factors, a single gene itself, and its interaction with E factors is fitted. Specifically, the conceptual marginal model is “Outcome ~ Es + G + G*(Es)”, where the outcome variable can be a continuous, categorical, or survival time phenotype, Es represents a set of environmental factors such as environmental exposures, demographic, clinical, and socioeconomic variables, and G*(Es) represents the interaction between the G factor and all E factors under consideration. The significant G-E interactions can be selected based on the corresponding marginal *p*-values. Since the marginal model is low-dimensional, its main advantage is its computational stability and conceptual simplicity. Therefore, marginal programs are popular in the fields of bioinformatics and biomedicine. However, a common limitation of traditional methods of marginal analysis is its lack of robustness. In practical genetic studies, Xu et al. [5] pointed out that long-tailed distributions and contamination in prognosis response and predictors are not uncommon. In addition, human input errors may also lead to long-tailed distributions and contamination. In Fig. 1, we displayed The Cancer Genome Atlas (TCGA) clinical survival data for esophageal carcinoma (ESCA) and head and neck squamous cell carcinoma (HNSCC) to show the long-tailed distribution phenomenon. Moreover, censored survival outcomes make the relevant feature screening difficult.

On the other hand, models in the framework of joint analysis better describe disease biology given the fact that complex diseases are related to the combined effects of multiple genetic biomarkers. The conceptual joint model is “Outcome ~ Es + Gs + (Gs)*(Es)”, where Gs represents a set of G factors, including gene expressions, SNPs and other types of molecular measurements, and (Gs)*(Es) represents the interactions between all G and E factors. In this article, we focus on the joint analysis framework. A common challenge of joint analysis is its high dimensionality, which makes it difficult to identify significant interaction effects. Moreover, right-censored survival outcomes and contaminated biomarker data make the task even challenging.

For survival outcomes, popular models include the accelerated failure time (AFT) model and Cox’s model. Based on the AFT model, several robust joint regression methods have been proposed. The Penalized trimmed regression (PTReg) method [6] uses the trimmed regression to account for long-tailed distribution/contamination in prognosis response and predictors, and Wu et al. [7] incorporates the G structure into the joint modeling. These methods conduct regularized estimation and selection based on the minimax concave penalty (MCP) penalty and utilize a decomposition technique to explain the interaction hierarchy. Their main potential disadvantage is that the model size is much larger than the sample size, and the statistical power under the penalized regression frameworks may be suboptimal [8]. In addition, since the gene expression data is often contaminated, the traditional Pearson correlation or Gaussian graphical models may not be a suitable measure to quantify the correlation among genes [9].

Based on the above rationale, we plan to adopt a two-step screening approach to detect G-E interactions by incorporating biological pathways information. The proposed method uses annotated gene sets collected in the molecular signatures database [10], which can be downloaded from the website http://www.broadinstitute.org/gsea/msigdb. Wang and Chen [11] described the idea of an overlapping group screening procedure that aims at gene-gene interaction selection, called the OGS method, for survival prediction based on the Cox model. In this work, we extend and modified the OGS method to detect G-E interactions, and show that OGS has several advantages: (1) it can alleviate the collinearity problem in regression analysis due to the correlation between biomarkers in the same gene/pathway; (2) it can significantly reduce the search space for interaction effects by using the feature grouping structure; and (3) it can significantly improve the model selection performed by penalized regression in an ultra-high dimensional feature space.

Simulation studies under various scenarios reveal that our method works well and outperforms existing methods in the model selection, estimation, and prediction accuracy. In the real data application, we combine gene expression profile data with prior pathway information from the Gene Ontology biological process (GO-BP) database and use the OGS approach to select several important environmental factors, genes, and G-E interactions that are associated with clinical survival outcomes of patients with HNSCC, and ESCA using TCGA clinical survival genetic data [12]. Using the pathway information available from the GO-BP database to group genes into several pathways, we further conduct accurate survival predictions based on the selected main and interacting biomarkers.

## Methods

We consider a study with $N$ independent subjects. For a subject ${\text{i}}$, suppose that there are $q$ environmental/clinical variables ${\varvec{e}}_{i} = \left( {e_{i1} , \cdots ,e_{iq} } \right)^{^{\prime}}$, and $p$ genes ${\varvec{x}}_{{\varvec{i}}} = \left( {x_{i1} , \cdots ,x_{ip} } \right)^{^{\prime}}$ assigned to $G$ possibly overlapping pathways; that is, a given gene may belong to multiple pathways. The pathway information accounts for the natural hierarchical structure of genes, and the overlapping pathways commonly exist in the gene expression data. Our aim is to determine the main features (genes and environment) and their interactions related to clinical survival outcomes, while taking into account the pathway information.

For a subject $i$, assume the survival outcome $t_{i}$ is related to the environmental/clinical variables ${\varvec{e}}_{i}$, gene expression covariates ${\varvec{x}}_{{\varvec{i}}}$, and their component-wise interactions ${\varvec{u}}_{i} = \left( {e_{i1} x_{i1} , \ldots , e_{i1} x_{ip} , e_{i2} x_{i1} , \ldots , e_{iq} x_{ip} } \right)^{^{\prime}}$ through the Cox regression model. In the Cox regression framework, the hazard function at time $t$ for subject $i^{\prime}{\text{s}}$ survival given the covariates is modeled as.

$\lambda \left( {t|{\varvec{e}}_{i} , {\varvec{x}}_{i} ,{\varvec{u}}_{i} } \right) = \lambda_{0} \left( t \right)exp\left( {{\varvec{e}}_{i}^{^{\prime}} {\varvec{\alpha}} + {\varvec{x}}_{i}^{\user2{^{\prime}}} {\varvec{\beta}} + {\varvec{u}}_{i}^{\user2{^{\prime}}} {\varvec{\eta}}} \right)$,where $\lambda_{0} \left( t \right)$ is a non-negative deterministic baseline hazard function and $\left( {{\varvec{\alpha}}, {\varvec{\beta}},{\varvec{\eta}}} \right)$ are corresponding parameters. Usually the survival outcome is subject to censoring, and we use $\delta_{i}$ to denote whether subject $i^{\prime}{\text{s}}$ survival time is observed or censored.

Incorporating the grouping (pathway) information into the modeling process may improve the interpretability and prediction accuracy of the model. When groups overlap with each other, special techniques are required to account for the overlapping grouping information. According to Jacob et al. [13], we decompose the original coefficient vector into the sum of group-specific potential effects, that is, ${\varvec{\beta}} = \mathop \sum \limits_{j = 1}^{G} {\varvec{\gamma}}^{j}$ where ${\varvec{\gamma}}^{j} = \left( {\gamma_{1}^{j} , \cdots ,\gamma_{p}^{j} } \right)^{^{\prime}}$ is the latent coefficient vector for group $j$. For $j = 1, \ldots ,G$ and $k = 1, \ldots ,p$, we set $\gamma_{k}^{j} = 0$ if gene k does not belong to group $j$. Redefine the latent coefficient ${\varvec{\gamma}}^{j}$ by removing the zero elements therein, and form the latent coefficient vector ${\varvec{\gamma}}$ by stacking the vectors ${\varvec{\gamma}}^{1} , \ldots , {\varvec{\gamma}}^{G}$. Let $d$ be the length of $\user2{ \gamma }$. We can then rewrite ${\varvec{\beta}} = \user2{S\gamma }$, where ${\varvec{S}}$ is a $p \times d$ matrix whose elements are 1 or 0. A simple example for illustration is given in Additional file 1: Appendix S1.

On the basis of the coefficient decomposition, the original regression model can be transformed into a new model, that is,${\varvec{X}}_{N \times p} {\varvec{\beta}}_{p \times 1} = {\varvec{X}}_{N \times p} {\varvec{S}}_{p \times d} {\varvec{\gamma}}_{d \times 1} = \tilde{\user2{X}}_{N \times d} {\varvec{\gamma}}_{d \times 1}$, where ${ }{\varvec{X}} = \left( {{\varvec{x}}_{1} , .{ }.{ }.{ },{\varvec{x}}_{N} } \right)^{^{\prime}}$. Equivalently, this new model can be constructed by duplicating the columns of overlapping variables in the original design matrix. For the new transformed model, the hazard function for subject $i$ in the Cox regression model is re-expressed as

$$\lambda \left( {t|{\varvec{e}}_{i} ,\tilde{\user2{x}}_{i} ,{\varvec{u}}_{i} } \right) = \lambda_{0} \left( t \right)exp\left( {{\varvec{e}}_{i}^{^{\prime}} {\varvec{\alpha}} + \tilde{\user2{x}}_{i}^{^{\prime}} {\varvec{\gamma}} + {\varvec{u}}_{i}^{^{\prime}} {\varvec{\eta}}} \right)$$

### The method (OGS) for G-E interaction selection

We apply the OGS method to the environment and gene expression profile data with clinical survival trait to detect important main effects as well as interactions by incorporating prior pathway information. The steps of the OGS algorithm for G-E interaction selection are described as follows.

*Step1* We utilize the overlapping group Cox regression model to identify the candidate pathways based on the latent effect approach, which can be performed by the R package “*grpregOverlap*” [14]. We define $\hat{M}_{main}$ as the selected set of pathways, and $A = \left| {\hat{M}_{main} } \right|$ as the size of $\hat{M}_{main}$.

*Step 2* We utilize the sequence kernel association test (SKAT) to obtain the group-specific significance, where each group is formed by the interaction between the genes of each candidate pathway selected in the first step and the environmental factors in Es, where Es is a set of environmental factors. Following Chen et al. [15], the SKAT statistic under the Cox regression model is defined as

$$Q_{k} = \user2{m^{\prime}R}_{\left( k \right)} {\varvec{W}}_{\left( k \right)} {\varvec{W}}_{\left( k \right)} {\mathbf{R}}_{{\left( {\text{k}} \right)}}^{^{\prime}} {\varvec{m}}, k = 1, \ldots ,A$$

Here ${\varvec{m}}$ is the vector of martingale residuals estimated from the null model by regressing survival outcomes on only the environmental covariates Es without considering the gene expression data; ${\varvec{R}}_{\left( k \right)} = \left[ {r_{\left( k \right)ij} } \right]_{N \times l}$, where $l$ is the number of G-E interaction pairs in the candidate pathway group $k$, $r_{\left( k \right)ij}$ is the $j$-th G-E interaction pair of $i$-th subject in the candidate pathway group $k$, and ${\varvec{W}}_{\left( k \right)}$ is a diagonal weight matrix that contains the weights of the $l$ interaction pairs in the candidate pathway group $k$. Suitable weights can improve the testing power [16]. Following [16], we consider an unsupervised weight manner that is defined as

$$\sqrt {{\varvec{W}}_{\left( k \right)i,i} } = Beta\left( {v_{i} ,\;1,\;25} \right),\;i = 1, \ldots l;k = 1, \ldots ,A$$

where $v_{i} = \frac{{Var\left( {r_{\left( k \right) \cdot i} } \right)}}{{\mathop \sum \nolimits_{j} Var\left( {r_{\left( k \right) \cdot j} } \right)}}$. That is, the square of the weight is a beta probability density function with specific parameters 1 and 25, evaluated at the ratio of the sample variance of the *i-th* variable in the data to that of all variables.

Based on the null model by regressing survival outcomes on only the environmental covariates Es without gene covariates, let ${\varvec{E}}$ is an $N \times q$ design matrix for the $q$ environmental covariates, and ${\varvec{V}} = {\text{diag}}\left( {c_{1} , \ldots ,c_{N} } \right) - {\varvec{PP}}^{^{\prime}}$, where ${\varvec{P}}$ is an $N \times \nu$ matrix with element $p_{ij}$ the baseline hazard for individual $i$ at ordered failure time $t_{\left( j \right)}$,${ }j = 1, \ldots ,\nu ,$ and $c_{i}$ the cumulative hazard for individual $i$ at observed time $t_{i}$.

Let $\sum_{\left( k \right)} = {\varvec{W}}_{\left( k \right)} {\varvec{R}}_{\left( k \right)}^{^{\prime}} \left( {{\varvec{V}} - {\varvec{VE}}\left( {\user2{E^{\prime}VE}} \right)^{ - 1} {\varvec{E}}^{^{\prime}} {\varvec{V}}} \right){\varvec{R}}_{\left( k \right)} {\varvec{W}}_{\left( k \right)}$ be the covariance matrix of the vector ${\varvec{W}}_{\left( k \right)} {\varvec{R}}_{\left( k \right)} {\varvec{m}}$ under the null hypothesis of all gene-environment interaction pairs in the candidate pathway group $k$ having null effects. Under the null hypothesis, the SKAT statistic follows a weighted sum of chi-square distribution:

$$Q_{\left( k \right)} \sim \mathop \sum \limits_{j = 1}^{l} \lambda_{\left( k \right)j} \chi_{1,j}^{2} ,$$

where $\lambda_{\left( k \right)j}$*,j* $=$ *l,…,* $l$ are the eigenvalues of $\sum_{\left( k \right)}$, and $\chi_{1,j}^{2}$'s are independent 1-df central chi-square random variables.

We use the Davies method [17] to approximate the tail probability of the mixture chi-square distribution, which can be calculated by the R package “*CompQuadForm*” [18]. Generally speaking, the Davies method is accurate [19]. The *p*-values $\left\{ {p_{1, \ldots ,} } \right.\left. {p_{A} } \right\}$ are used as our group screening measure; a smaller *p*-value corresponds to a higher group importance and therefore leads to a higher priority of selection.

*Step 3* In the third step, we select significant G-E interactions based on the permutation procedure with the cutoff point determined by the soft-thresholding rule, where the permutation is applied to the covariate matrix consisting of both genes and environmental covariates. We randomly permute the original data $\left\{ {Y_{i} ,{\varvec{e}}_{i} } \right.,\left. {{\varvec{x}}_{i} } \right\}$ to form the permuted data.

$\left\{ {Y_{i} ,{\varvec{e}}_{\pi \left( i \right)} } \right.,\left. {{\varvec{x}}_{\pi \left( i \right)} } \right\}$ following the null model, where $Y_{i} = \left( {t_{i} ,\delta_{i} } \right)$ is the survival outcome, and ${ }\left\{ {\pi \left( 1 \right),. . .,\left. {\pi \left( N \right)} \right\}} \right.$ is a random permutation of the index. Then we apply again the SKAT test for each of the candidate pathway groups with the permuted data to obtain the group screening measures (*p*-values) $\left\{ {p_{1}^{*} } \right.,. . .,\left. {p_{A}^{*} } \right\}$ and the desired threshold $\tau$ is obtained by taking the minimum of $\left\{ {p_{1}^{*} } \right.,. . .,\left. {p_{A}^{*} } \right\}$.

To obtain a stable threshold, we repeat the above permutation process more times and define a cutoff point to select candidate pathway groups by using the median of the obtained desired thresholds, that is $C_{int} =$ median $\left\{ {\tau_{1} } \right.,. . .,\left. {\tau_{I} } \right\}$. We adopt $C_{int}$ to select candidate pathway groups, i.e.

$$\hat{M}_{int} = \left\{ {b: p_{b} < } \right.C_{int} ,b = 1,. . . ,\left. A \right\},$$

is our selected set of candidate pathway groups. In practice, we take ${ }I$ as 30.

Note that he permutation procedure used to determine a data-driven threshold was similar to that proposed by Fan et al. [20], which implicitly assumes that the censoring mechanism is independent of all covariates. This stronger assumption on censoring mechanism will not invalidate the permutation procedure since what we indeed require for the null hypothesis is that the tuple of time and censoring indicator is independent of all the covariates.

*Step 4* Finally, in the framework of joint modeling, based on environmental covariates, and selected genes and G-E interactions, a penalized regression with an appropriate penalty is used to establish the final survival prediction model. Therefore, we apply the penalized Cox’s regression together with the Ridge or Lasso penalty to build the final prediction model based on all environmental variables, genes in ${ }\hat{M}_{main}$ and G-E interactions in $\hat{M}_{int}$. The penalized Cox regression model with the Ridge or Lasso penalty can be obtained through the R package “*glmnet*” [21].

In the first step and the second step of the new OGS method, as the original OGS method in Wang and Chen [11], we still apply the overlapping group selection method to identify the causal pathways and the SKAT test to obtain the group-specific significance. However, the new OGS method improves the original one [11] by using an unsupervised manner for weight construction in the second step of the OGS procedure. In the third step of the new OGS method, we perform multiple permutations to obtain a stable threshold for interaction group selection, where the permutation process is the same as in the original OGS, except that permeation is now applied to a covariate matrix consisting of genes and environmental factors. Finally, the penalized Cox’s regression with the Ridge or Lasso penalty is still applied to build the final survival prediction model based on the environmental factors, the selected genes and the selected G-E interactions. These modifications bring better performance for model selection, estimation, and prediction.

#### Results

### Comparison with alternative methods in variable selection, estimation, and prediction

In the following simulations, we study the performances of the proposed OGS approach in variable selection, estimation and prediction, and compare them with the performance of the “Oracle”, “SIS Lasso”, “Ordinary Lasso” and “GSIS SCAD” methods. The “Oracle” method is based on the underlying true model, which is known in the simulations but unknown in real applications. The “SIS Lasso” method [8] uses univariate Cox’s regression to select environmental variables, genes, and G-E interactions one by one, with a prefixed number $\left( {\frac{N}{\log \left( N \right)}} \right)$ of top-ranked predictors as our candidate model, and then includes the selected variables in a penalized Cox regression model with the Lasso penalty to form the final prediction model. The “Ordinary Lasso” method is the penalized Cox regression model with the Lasso penalty considering all environmental variables, genes, and G-E interactions in the model. The “GSIS SCAD” method is an overlapping group Cox regression model with the SCAD penalty based on the latent effect approach, which can be performed by the R package “*grpregOverlap*” [14].

For performance comparison, we adopt the root mean squared error (RMSE) to measure estimation accuracy, defined as

$$RMSE = \sqrt {\frac{1}{S}\mathop \sum \limits_{j = 1}^{S} \left( {\theta_{j} - \widehat{{\theta_{j} }}} \right)^{2} }$$

where $S$ is the size of the full model including all main and interaction covariates and ${\varvec{\theta}}^{\prime } = \left( {{\varvec{\alpha}}^{\prime } ,{\varvec{\beta}}^{\prime } ,{\varvec{\eta}}^{\prime } } \right)$.

To evaluate the estimation performance, we report RMSE.M, the mean of the root mean square errors of 200 simulations. To evaluate the performance of the selection accuracy, we consider various criteria: P.int is the proportion of the underlying effective G-E interaction variables contained by the selected G-E interaction variables; Sen. is the sensitivity, defined as the proportion of the underlying effective variables being selected; Spe. is the specificity, defined as the proportion of the underlying ineffective variables not being selected. We also report the median size of the selected models, S.model, in 200 simulations. To evaluate the performance of survival prediction, we consider three measures of prediction accuracy: the deviance, the c-index proposed by Harrell et al. [22] and time-dependent AUC proposed by Blanche et al. [23]; smaller deviance or larger c-index and time-dependent AUC corresponds to better prediction accuracy. The median values of these measure over 200 simulations are reported.

Let $\hat{\user2{\theta }}^{\user2{^{\prime}}} = \left( {\hat{\user2{\alpha }}^{\user2{^{\prime}}} ,\hat{\user2{\beta }}^{\user2{^{\prime}}} ,\hat{\user2{\eta }}^{\user2{^{\prime}}} } \right)$ an estimator of the (penalized) Cox regression parameter in a prediction model obtained from the training dataset. Let ${ }\left( {t_{i}^{\user2{*}} ,\delta_{i}^{*} ,{\varvec{e}}_{i}^{\user2{*}} ,{\varvec{x}}_{i}^{\user2{*}} ,{\varvec{w}}_{i}^{\user2{*}} } \right)$ be the survival and covariate data of subject $i{ }$ in the test data. Define $\left( {{\varvec{e}}_{i}^{*\prime } ,{\varvec{x}}_{i}^{*\prime } ,{\varvec{w}}_{i}^{*\prime } } \right)\hat{\user2{\theta }}$ as the prognosis index (PI) value for subject $i$ in the test data. The Cox test is defined as the *p*-value of PI when PI is used as the covariate in the univariate Cox model for survival outcomes in the test data. Similarly, the LR-test is the *p*-value of the log-rank test for the null hypothesis of equal survival between the “good” and “poor” prognostic groups in the test data, where the “good” and “poor” prognostic groups are classified according to whether the PI value is higher or lower than the median PI value in the test data. Smaller Cox-test and LR-test values correspond to better predictive power.

In simulations we consider survival data with a cohort size 300 in the training set, where each subject’s survival time follows the Cox proportional hazards model

$$\lambda_{0} \left( {t|{\varvec{e}}, {\varvec{x}},{\varvec{w}}} \right) = 10exp\left( {\user2{e^{\prime}\alpha } + \user2{x^{\prime}\beta } + \user2{w^{\prime}\eta }} \right),$$

with the covariates ${\text{e}}$ and ${\varvec{x}}$ jointly following a multivariate standard normal distribution with correlation $corr\left( {{\varvec{e}}_{ \cdot j} ,{\varvec{e}}_{ \cdot k} } \right) = 0.3^{{\left| {j - k} \right|}}$ and $corr\left( {{\varvec{x}}_{ \cdot j} ,{\varvec{x}}_{ \cdot k} } \right) = 0.5^{{\left| {j - k} \right|}}$, and $corr\left( {{\varvec{e}}_{ \cdot j} ,{\varvec{x}}_{ \cdot k} } \right) = 0$ for all $j, k$. The censoring time distribution follows a uniform distribution. We then generate survival data, independent of the training data, with a cohort size 100 as the test data to assess the prediction accuracy for different methods.

**Fig. 2:**
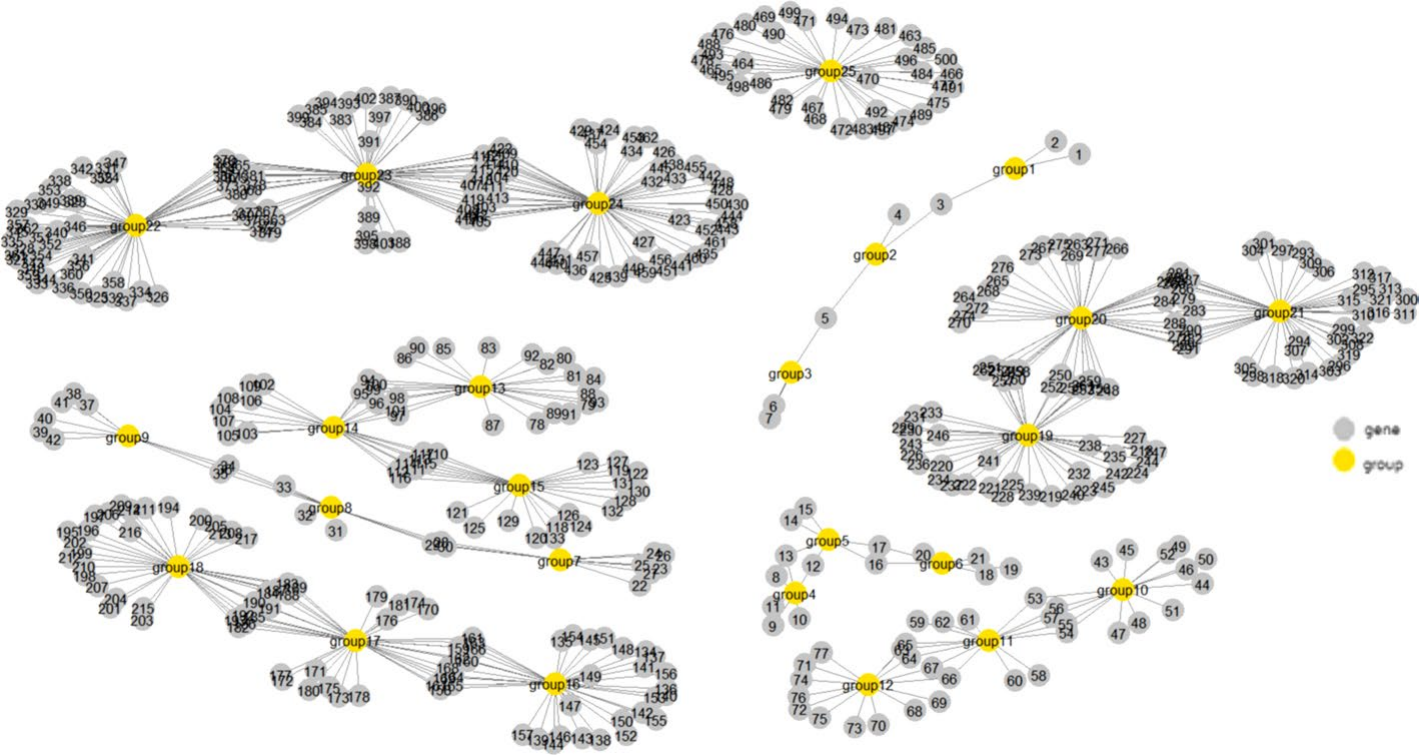
Gene network structure

In this simulation study, we consider 5 environmental variables and assume that the first 4 are related to the survival outcome, and the corresponding effects are 1.5, 2.25, 3, -1.5. On the other hand, the gene covariates considered contain 25 groups that have different group sizes (the numbers of genes) and may share with each other some of the genes. The group sizes and the overlapping structure (i.e. the number of the shared genes between two overlapping groups) are shown in Table 1, where the overlapping groups are shown side by side. For example, group 1 contains 3 genes, as group 2 does, but the two groups contain only 5 unique genes, and 1 gene is shared between the two groups. As a result, there are a total of 500 genes and 632 group-specific latent effects (see “Methods” section) in this example. Figure 2 displays the gene network structure. Groups 1, 7, 13, and 19 are set to be effective, and genes in each of them have constant latent effects of 3, 3, 2, and − 2, respectively. In addition, effective interactions (E1 * G22, E1 * G24, E2 * G26) with the corresponding effects $\left( {1.5,1.5,2} \right)$ and (E2 * G78, E3 * G83, E3 * G88) with the corresponding effects $\left( { - 1, - 1.5, - 2} \right)$ are in group 7 and group 13, respectively. The number of effective environment, gene, or G-E interaction factors is 91 among a total of 3,005 such factors. We examine the performances of different methods under a censoring rate of 30%, 50%, or 70%. We also conduct further simulations to demonstrate the performances of the new proposal, whose details and results can be seen in Additional file 1: Appendix S2.

**Table 1:** Gene group structure in the simulation study

Pathway	1	2	3	4	5	6	7	8	9	10	11	12	13	14	15	16	17	18	19	20	21	22	23	24	25
Gene Size	3	3	3	6	6	6	9	9	9	15	15	15	24	24	24	36	36	36	45	45	45	60	60	60	38
Overlapping		1	1	0	2	2	0	3	3	0	5	5	0	8	8	0	12	12	0	15	15	0	20	20	0

### Summary of simulation results

From the simulation results shown in Table 2 and Additional file 1: Table S3 in Appendix S2 where the gene network structure is complex, we see that the OGS method using the Lasso or Ridge penalty performs substantially better than the “SIS Lasso”, “Ordinary Lasso” and “GSIS SCAD” methods in variable selection, effect estimation, and survival prediction. On the other hand, simulation results shown in Additional file 1: Table S3 of Appendix S2 where a simpler gene structure is considered, the performance of OGS with Lasso or Ridge penalty is worse than that of the “Ordinary Lasso” method when the censoring rate is 30%; while when the censoring rate is higher (50% or 70%), the OGS with the Lasso or Ridge penalty performs better than the “Ordinary Lasso”.

**Table 2:** The median of the performance measures out of 200 simulation replications for different approaches

	Oracle	GSIS SCAD	SIS lasso	Ordinary lasso	OGS ridge	OGS lasso
*Censoring rate = 30%*						
RMSE	0.3520	0.2936	0.3629	0.3582	0.3667	0.3194
P.int	1.0000	0.0000	0.0000	0.1667	0.5000	0.5000
Sen	1.0000	0.8901	0.3736	0.7473	0.9670	0.9670
Spe	1.0000	1.0000	0.9962	0.9823	0.9399	0.9875
C.model	91.0000	81.0000	46.0000	120.0000	266.0000	124.0000
Deviance	− 125.2257	− 113.7699	− 60.0277	− 114.7706	− 70.2329	− 250.4203
C-index	0.8727	0.9244	0.7875	0.8722	0.8969	0.9549
AUC	0.9392	0.9730	0.8540	0.9418	0.9650	0.9908
*Censoring rate = 50%*						
RMSE	0.3437	0.3668	0.3631	0.3618	0.3670	0.3451
P.int	1.0000	0.0000	0.0000	0.1667	0.5000	0.3333
Sen	1.0000	0.8901	0.3516	0.5934	0.9670	0.8791
Spe	1.0000	1.0000	0.9955	0.9825	0.9221	0.9911
C.model	91.0000	81.0000	45.0000	104.5000	311.0000	104.0000
Deviance	− 96.4789	− 32.3585	− 43.5888	− 66.1335	− 50.0469	− 132.8876
C-index	0.8841	0.8027	0.7915	0.8491	0.8929	0.9222
AUC	0.9363	0.8537	0.8433	0.8985	0.9461	0.9668
*Censoring rate = 70%*						
RMSE	0.3407	0.3671	0.3643	0.3654	0.3674	0.3561
P.int	1.0000	0.0000	0.0000	0.0000	0.5000	0.1667
Sen	1.0000	0.8901	0.2747	0.4176	0.9670	0.6484
Spe	1.0000	1.0000	0.9942	0.9849	0.9224	0.9935
C.model	91.0000	81.0000	43.0000	83.0000	294.5000	77.0000
Deviance	− 0.8763	− 16.1453	− 22.9206	− 31.5497	− 31.7002	− 58.9638
C-index	0.8617	0.7569	0.7791	0.8174	0.8837	0.8866
AUC	0.8996	0.7844	0.8136	0.8430	0.9201	0.9212

Furthermore, further simulation studies with a small cohort size are conducted under the scenario where all simulated settings are the same as those in the previous simulation study except for a cohort size defined as 150/50 (training/testing). We still obtain similar numeric results patterns; these corresponding results are shown in Additional file 1: Tables S5–S7 in Appendix S3. In addition, we also conduct a simulation study that the overlapping genes occur among three groups instead of just two, where we still obtain result patterns similar to those under two groups; please see Additional file 1: Table S8 in Appendix S2.

### Real data application: TCGA HNSCC data

The TCGA HNSCC RNA-Seq expression data, together with the phenotype data containing the survival time and censoring status data, can be downloaded from the R package’TCGAbiolinks’ [24], or’UCSCXenaTools’ [25]. After excluding patients with missing survival time data, our analysis is focused on the subset of the TCGA HNSCC data with 517 patients and 20,501 gene expression variables. The censoring rate of the survival time in the data is about 58%. The TCGA HNSCC clinical information data can be obtained from the’FireBrowse’ database [26].

Since the number of cancer-related genes is expected to be limited, we conduct prescreening using non-parametric inverse probability-of-censoring weighted (IPCW) Kendall’s tau correlation [27], which can also improve stability for feature selection. The top 2000 genes with the largest absolute IPCW Kendall’s tau correlation are selected for downstream analysis.

The five E factors analyzed including AJCC pathologic stage nodes, AJCC pathologic stage tumor, age, gender, and ICD O3 site. Summary information for these clinical variables is reported in the Table 3. Some of the clinical variables contain missing values, and we use the sparse boosting method [28] in the R package “*GEInter*” [29] to perform multiple imputation for the missing values in the clinical variables.

**Table 3:** The selected clinical variables information of the TCGA HNSCC

Variable	Coding	Missing status	Continuous(EC) /discrete(ED)
AJCC pathologic nodes	n0 = 0, n1 = 1, (n2, n2a, n2b, n2c) = 2, n3 = 3, nx = 4	YES	ED
AJCC pathologic tumor	t0 = 0, t1 = 1, t2 = 2, t3 = 3, (t4, t4a, t4b) = 4, tx = 5	YES	ED
age		No	EC
gender	female = 0, male = 1	No	ED
ICD O3 site	(C00.9, C01.9, C02.1, C02.9) = 0, (C03.0, C03.1, C03.9, C04.0, C04.9) = 1, (C05.0, C05.9 C06.0, C06.2, C06.9) = 2, (C09.9, C10.3, C10.9) = 3, (C13.9, C14.8) = 4, and 5 for others	No	ED

The PTReg method [5] was developed to conduct robust joint analysis using penalized trimmed regression with the MCP penalty under the AFT model for the right-censored survival outcome. We are interested in comparing the PTReg approach with our proposed OGS approach in the real data application. The whole 12,005 main and G-E interaction predictors are considered for the “SIS Lasso”, “Ordinary Lasso”, and “PTReg” methods. For the OGS method, among the 2000 preselected genes, prior pathway information for 1489 genes, which are mapped into 6015 pathways based on the GO biological process database, is utilized. The 511 genes that are not mapped into any pathways in the GO biological process database are either discarded or put together as a group for the latent effect analysis in the OGS method, leading to a total of 8939 or 12,005 main and G-E interaction effects considered.

We take ten random splits of the whole data into 413:104 training/test sets to evaluate the performances of all the methods considered in the TCGA HNSCC data application. Table 4 reports the median of the survival prediction results over the ten folds when the 511 ungrouped genes are discarded from analysis. We see that the performance of the OGS method with Ridge or Lasso penalty is better than the “SIS Lasso”, “Ordinary Lasso”, and “PTReg” methods. The OGS approach putting the 511 ungrouped genes together as an additional group results in the same prediction model as the one discarding the ungrouped genes. Also, the OGS analysis results based on the pathway information obtained from other annotated gene set databases, including GO cellular component (GO-CC), GO molecular function (GO-mf), and KEEG, are compared with the other methods for survival prediction in the TCGA HNSCC data, as shown in Additional file 1: Table S9. These additional results based on pathway information from alternative gene set databases still reveal that the OGS approach performs better than the other methods.

**Table 4:** Results (median of prediction accuracy of different methods in the TCGA HNSCC data over 10 random splits of 413:104 training /test sets based on GO-BP database)

	GSIS SCAD	SIS lasso	Ordinary lasso	OGS ridge	OGS lasso	PTReg
Cox-test	0.1842	0.0048	0.0029	0.0002	0.0013	0.0660
LR-test	0.2949	0.0115	0.0117	0.0015	0.0077	0.0580
Deviance	34.6441	8.7698	2.8340	0.0899	2.8927	44.9984
C-index	0.5534	0.6323	0.6471	0.7066	0.6618	0.5851
AUC	0.5231	0.6505	0.6432	0.7005	0.6660	0.6213

**Fig. 3:**
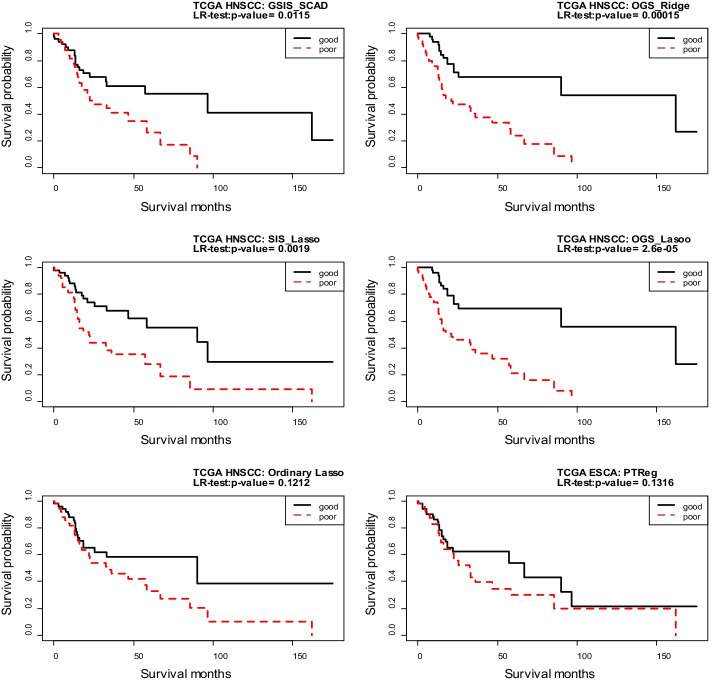
Kaplan–Meier curves for the 104 subjects in the TCGA HNSCC testing data. Good and poor groups are identified by the median of the PI scores in the test dataset

Based on one random split of the data, Fig. 3 displays the Kaplan–Meier survival curves of the “good” and “poor” prognosis groups in the test data. It can be seen that the OGS method separates the two groups better than other methods. When applying the OGS with the Lasso penalty to the entire data based on the GO biological process database, we identify several important G-E interaction effects, and obtain the corresponding parameter estimates, as shown in Additional file1: Table S10. We note that the clinical variable “Age” interacts with several genes, and most of these genes, such as “*CAMP*” [30], “*DEFB1*” [31], “*MAP2K7*” [32] have been shown to be related to HNSCC. And “Age” factor has been shown to be related to HNSCC [33].

### Real data application: TCGA ESCA data

The TCGA ESCA RNA-Seq expression data, together with the phenotype data containing the survival time and censoring status data, can be downloaded from the R package’TCGAbiolinks’ [24], or’UCSCXenaTools’ [25]. After excluding patients with missing survival time data, our analysis is focused on the subset of the TCGA ESCA data with 368 patients and 20,501 gene expression variables. The censoring rate of the survival time in the data is about 58%. The TCGA ESCA clinical information data can be obtained from the’FireBrowse’ database [26].

Since the number of cancer-related genes is expected to be limited, we conduct prescreening using non-parametric inverse probability-of-censoring weighted (IPCW) Kendall’s tau correlation [27], which can also improve stability for feature selection. The top 2000 genes with the largest absolute IPCW Kendall’s tau correlation are selected for downstream analysis. The seven clinical variables whose E effects are analyzed include age, gender, esophageal tumor central location, person neoplasm cancer status, race, BMI, and AJCC pathologic stage, and their summary information is reported in the Table 5. Some of the clinical variables contain missing values, and we use the sparse boosting method in the R package “*GEInter*” to perform multiple imputation for the missing values in the clinical variables. Based on the GO biological process database, 1458 genes among the top 2000 genes are mapped into 4360 pathways and such prior pathway information is utilized in the OGS method. Excluding the genes without being mapped into any pathway, there are a total of 11,671 main and G-E interaction covariates in the proposed OGS method. On the other hand, a total of 16,007 main and G-E interaction predictors are considered in the “SIS Lasso”, “Ordinary Lasso”, and “PTReg” methods.

**Table 5:** The selected clinical variables information of the TCGA ESCA data

Variable	Coding	Missing status	Continuous(EC) /discrete(ED)
Esophageal tumor central location	proximal = 1, mid = 2, distal = 3	Yes	ED
Person neoplasm cancer status	tumor free = 1, with tumor = 2,	Yes	ED
Race	white = 1, asian = 2, black or African american = 3	Yes	ED
BMI	weight/height^2	Yes	EC
AJCC pathologic stage	(stage i, stage ia, stage ib) = 1 (stage ii, stage iia, stage iib) = 2 (stage iii, stage iiia, stage iiib, stage iiic) = 3 (stage iv, stage iva) = 4	Yes	ED
Age	days_to_ birth	No	EC
Gender	female = 0, male = 1	No	ED

We take ten random splits of the whole TCGA ESCA data into 294:74 training/test sets to evaluate the performances of all methods for survival prediction in the TCGA ESCA data. Table 6 reports the median of the survival prediction results among the ten folds. We see that the performance of the OGS method with the Ridge or Lasso penalty is better than the “SIS Lasso”, “Ordinary Lasso”, and “PTReg” methods. In addition to the OGS analysis discarding the 542 genes without mapped pathways in the GO biological process database, we also perform the OGS analysis putting the unmapped genes together as an additional group, and the two different implements of the OGS method result in the same prediction model. Also, different annotated gene sets databases, including GO-CC, GO-MF, and KEEG, are also used in the OGS approach to catch pathway information. As shown in Additional file 1: Table S11. the OGS method still outperforms than the other methods using such alternative pathway information.

**Table 6:** Results (median of prediction accuracy of different methods in the TCGA ESCA data over 10 random splits of 294:74 training /test sets based on GO-BP database)

	GSIS SCAD	SIS lasso	Ordinary lasso	OGS ridge	OGS lasso	PTReg
Cox-test	0.4685	0.0024	8.2557e − 09	6.0168e − 10	8.0676e − 10	0.0330
LR-test	0.4944	0.0308	6.1948e − 08	1.8792e − 08	1.2942e − 07	0.0244
Deviance	161.1422	11.4386	− 31.7249	− 44.0441	− 41.3946	57.3278
C-index	0.5452	0.6400	0.8759	0.8984	0.8862	0.7041
AUC	0.4843	0.5968	0.9006	0.9294	0.9109	0.7899

**Fig. 4:**
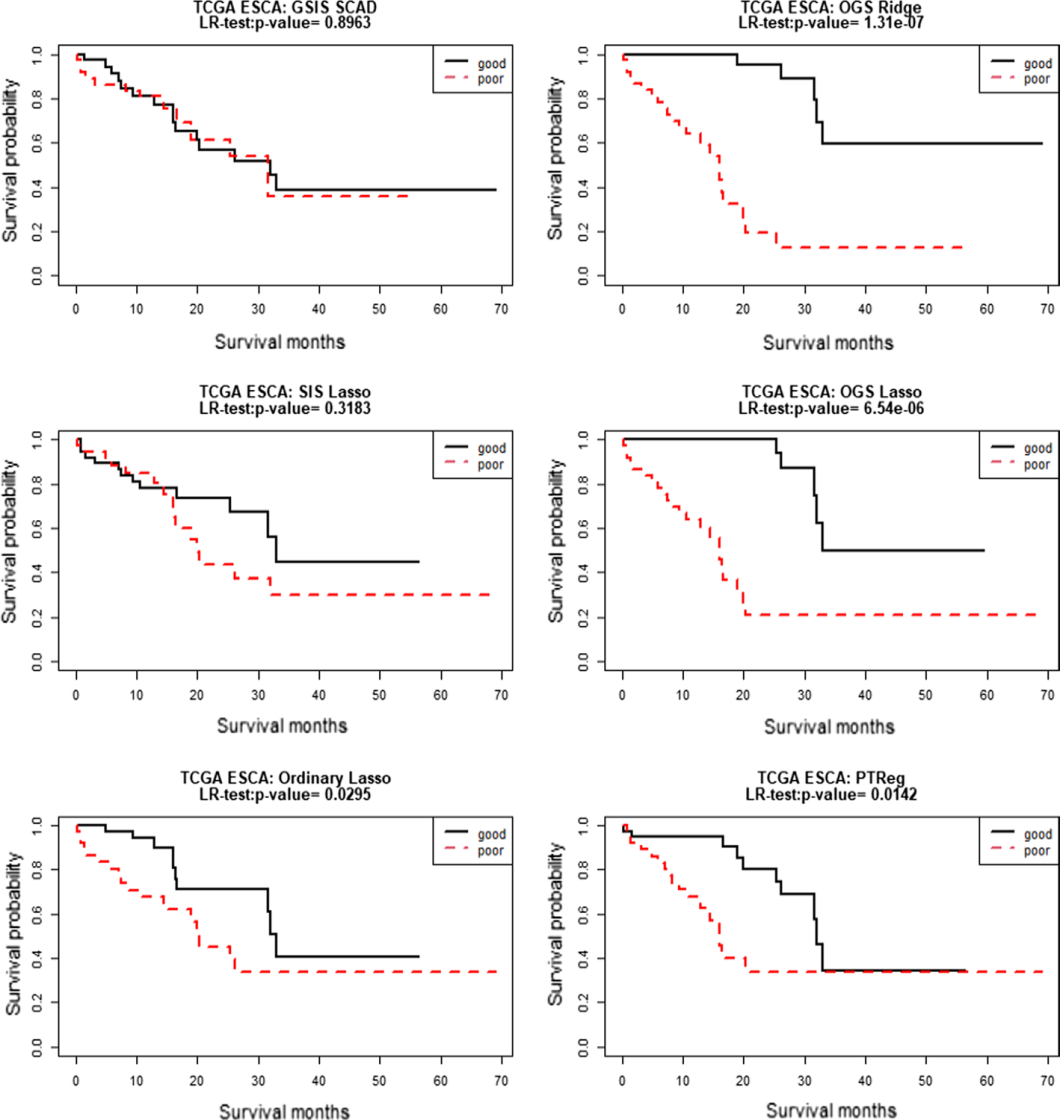
Kaplan–Meier curves for the 74 subjects in the TCGA ESCA testing data. Good and poor groups are identified by the median of the PI scores in the test dataset

Based on one random split of the data, Fig. 4 displays the Kaplan–Meier survival curves for the “good” and “poor” prognosis groups in the test data. It is seen that the two survival curves are better separated by the OGS approach than other methods. When applying the OGS with the Lasso penalty for whole data based on the GO biological process database, we identify and estimate several important G-E interaction effects, which are shown in Additional file 1: Table S12. We note that the clinical variable “Age” interacts with several genes, and most of these genes, such as “*CD40LG*” [34], “*DEK*” [35], “*IL6*” [36] have been shown to be related to HNSCC. And two “Weight” and “Age” factors have been shown to be related to HNSCC ([37, 38]).

## Conclusion

In this article, we propose a two-stage overlapping group screening procedure to identify important main and gene-environment (G-E) interaction effects efficiently for survival prediction. In the first stage, the new proposal utilizes the latent effect approach to identify candidate gene pathways for survival prediction, adjusting for the E and G-E interaction factors. Different gene pathways are allowed to overlap with each other, i.e., to share common genes. In the second stage, we utilize the SKAT approach [15], which is a popular group testing approach, to obtain the group-level *p*-value of each candidate gene pathway as well as the associated G-E factors, adjusting for the E factors. A pathway as well as the associated G-E factors is then selected when their group-level *p*-value is smaller than the one under covariate (both G and E factors) permutation. The final survival prediction model is constructed by a Cox model based on the E factors, the selected gene pathways as well as the associated G-E factors, subject to the Ridge or Lasso penalty. Simulation and real data studies demonstrate that, compared with the analysis that ignores pathway information, the new proposal can significantly improve the accuracy of gene and gene-environment interaction selection, as well as the resulting survival predictions.

The new OGS method aims at gene-environment interaction, while the OGS in Wang and Chen [11] aims at gene–gene interaction. The new OGS method improves the original one [11] by using an unsupervised manner for weight construction in step 2 of the OGS procedure, and performing multiple permutations to obtain a stable threshold for interaction group selection in step 3 of the OGS procedure. These modifications bring better performance for model selection, estimation, and prediction.

## Discussion

The OGS method is flexible. Although we focus on survival prediction based on the Cox proportional hazards model, the same idea can straightforwardly apply to other outcome models, such as the proportional odds survival time model, the logistic regression model for binary outcomes, and the multinomial logistic regression model for multi-class outcomes. For example, the SKAT statistic involved in the OGS method can be modified simply by using the residuals from the alternative model under consideration.

Since the gene data is high-dimensional, following the conventional feature screening idea, the initial step of the OGS method is to use some univariate approach to screen gene variables for downstream analysis. Such a supervised screening procedure is common (e.g., Fan et al. [20], Xu et al. [6], and Xu et al. [5]) in literature, and is in fact conducted after splitting the whole sample into the training and testing subsamples. In other words, when we evaluate the prediction performance using the test sample, the effect of supervised feature screening procedure has been taken into account and the evaluation is fair. We use the nonparametric inverse probability of censoring weighted (IPCW) Kendall’s tau correlation [27] to select the top 2,000 genes for downstream analysis. The IPCW Kendall’s measure it can be applied to a wide range of survival models, and the Kendall’s tau measure is not influenced by outliers, which is a major concern in gene expression data where contaminated data are common.

As in Jacob et al. [13] and Zeng and Breheny [14], the latent effect model is indeed over-parameterized, and the effects of each gene decomposed into the pathways are latent and unobserved. Owing to this nature of over parametrization, the penalized regression (group lasso) method is needed and employed for parameter estimation. Using such over-parametrization and penalized regression techniques, it is helpful to identify group-specific effects from the original Cox model regression parameters.

The OGS method employs the latent effect approach to extract gene network structure information in terms of gene pathways. This requires a pre-designated gene group (pathway) structure and is limited to genes that can be assigned to at least one group (pathway). It is interesting to study how to relax these restrictions to improve the performance of feature selection and survival prediction in the presence some covariate network structure.

In fact, the OGS procedure does not respect the hierarchy between main and interaction effects. We agree with that, if the hierarchy principle can be incorporated, the accuracy of interaction selection may have improved strength. Wu et al. [7] utilize a decomposition technique to explain the interaction hierarchy, and such decomposition technique may be incorporated into the OGS procedure as a further extension.

Moreover, the OGS method does not select at both the pathway level and the gene level simultaneously. How to improve the OGS in selecting pathways and genes simultaneously will be investigated in our future work. The last step of the OGS is to apply penalized Cox regression together with Ridge or Lasso penalty to build the final prediction model, we can try to combine the other penalties like MCP, Adaptive Lasso to enhance the robustness of the estimation of the OGS (Jiang et al. [39], Ren et al. [40]). This issue will also be investigated in our future work.

## Supplementary Information

**Additional file 1:** The full detail of the latent effect approach, a series of simulation studies, simulated settings where some genes are shared by three groups, and the real data analysis.

## Data Availability

The TCGA ESCA, and HNSCC genomic data with survival traits and pathway information database analyzed during this study are all available at figshare website https://doi.org/10.6084/m9.figshare.16816654.v6. R codes for the simulation studies and real data are available at figshare website https://doi.org/10.6084/m9.figshare.16816303.v3.

## Abbreviations

AFT: Accelerated failure time
AJCC: American joint committee on cancer
AUC: Area under the curve
BMI: Body mass index
ESCA: Esophageal carcinoma
GEInter: Gene-environment interaction
G-E: Gene-environment
GO-BP: Gene ontology biological process
GO-CC: Gene ontology cellular component
GO-MF: Gene ontology molecular function
GSIS: Grouping sure independence screening
HNSCC: Head and neck squamous cell carcinoma
IPCW: Inverse probability-of-censoring weight
Lasso: Least absolute shrinkage and selection operator
LR-test: Log-rank test
MCP: Minimax concave penalty
OGS: Overlapping grouped screening
PTReg: Penalized trimmed regression
PI: Predictor index
RMSE: Root mean squared error
SCAD: Smoothly clipped absolute deviation
SIS: Sure independence screening
SKAT: Sequence kernel association test
SNPs: Single-nucleotide polymorphisms
TCGA: The cancer genome atlas
