# Supplementary material for: Overlapping group screening for detection of gene-environment interactions with application to TCGA high-dimensional survival genomic data
Source: BMC Bioinformatics. 2022 May 30;23:202. doi: 10.1186/s12859-022-04750-7 (PMC9150322; doi:10.1186/s12859-022-04750-7)
Supplement: Supplementary file 1 — Additional file 1: The full detail of the latent effect approach, a series of simulation studies, simulated settings where some genes are shared by three groups, and the real data analysis. [file 12859_2022_4750_MOESM1_ESM.docx]

Web-based Supplementary Materials for

” **Overlapping Group Screening for Detection of Gene-environment Interactions with Application to TCGA High-dimensional Survival Genomic Data**”

by

Jie-Huei Wang, Kang-Hsin Wang and Yi-Hau Chen

**Appendix S.****1: Latent effect approach**

Suppose that there are six genes that are involved in the five pathways, P1={g1, g2, g3}, P2={g2, g3, g4}, P3={g1, g3, g5}, P4={g3, g4, g5} and P5={g1, g6}, the original coefficient $\mathbf{\beta}$ can be decomposed as

$$\mathbf{\beta}$$

$=\left[ \beta_{1},\beta_{2},\beta_{3},\beta_{4},\beta_{5},\beta_{6} \right]^{/}$

$$=\left[ \begin{aligned} \begin{matrix} \gamma_{1}^{1} \\ \gamma_{2}^{1} \\ \gamma_{3}^{1} \\ \gamma_{4}^{1} \end{matrix} \\ \gamma_{5}^{1} \\ \gamma_{6}^{1} \end{aligned} \right]+\left[ \begin{aligned} \begin{matrix} \gamma_{1}^{2} \\ \gamma_{2}^{2} \\ \gamma_{3}^{2} \\ \gamma_{4}^{2} \end{matrix} \\ \gamma_{5}^{2} \\ \gamma_{6}^{2} \end{aligned} \right]+\left[ \begin{aligned} \begin{matrix} \gamma_{1}^{3} \\ \gamma_{2}^{3} \\ \gamma_{3}^{3} \\ \gamma_{4}^{3} \end{matrix} \\ \gamma_{5}^{3} \\ \gamma_{6}^{3} \end{aligned} \right]+\left[ \begin{aligned} \begin{matrix} \gamma_{1}^{4} \\ \gamma_{2}^{4} \\ \gamma_{3}^{4} \\ \gamma_{4}^{4} \end{matrix} \\ \gamma_{5}^{4} \\ \gamma_{6}^{4} \end{aligned} \right]+\left[ \begin{aligned} \begin{matrix} \gamma_{1}^{5} \\ \gamma_{2}^{5} \\ \gamma_{3}^{5} \\ \gamma_{4}^{5} \end{matrix} \\ \gamma_{5}^{5} \\ \gamma_{6}^{5} \end{aligned} \right]=\left[ \begin{aligned} \begin{matrix} \gamma_{1}^{1} \\ \gamma_{2}^{1} \\ \gamma_{3}^{1} \\ 0 \end{matrix} \\ 0 \\ 0 \end{aligned} \right]+\left[ \begin{aligned} \begin{matrix} 0 \\ \gamma_{2}^{2} \\ \gamma_{3}^{2} \\ \gamma_{4}^{2} \end{matrix} \\ 0 \\ 0 \end{aligned} \right]+\left[ \begin{aligned} \begin{matrix} \gamma_{1}^{3} \\ 0 \\ \gamma_{3}^{3} \\ 0 \end{matrix} \\ \gamma_{5}^{3} \\ 0 \end{aligned} \right]+\left[ \begin{aligned} \begin{matrix} 0 \\ 0 \\ \gamma_{3}^{4} \\ \gamma_{4}^{4} \end{matrix} \\ \gamma_{5}^{4} \\ 0 \end{aligned} \right]+\left[ \begin{aligned} \begin{matrix} \gamma_{1}^{5} \\ 0 \\ 0 \\ 0 \end{matrix} \\ 0 \\ \gamma_{6}^{5} \end{aligned} \right]$$

$$=\left[ \begin{aligned} \begin{matrix} 1,0,0,0,0,0,1,0,0,0,0,0,1,0 \\ 0,1,0,1,0,0,0,0,0,0,0,0,0,0 \\ 0,0,1,0,1,0,0,1,0,1,0,0,0,0 \\ 0,0,0,0,0,1,0,0,0,0,1,0,0,0 \end{matrix} \\ 0,0,0,0,0,0,0,0,1,0,0,1,0,0 \\ 0,0,0,0,0,0,0,0,0,0,0,0,0,1 \end{aligned} \right]\left[ \gamma_{1}^{1},\gamma_{2}^{1},\gamma_{3}^{1},\gamma_{2}^{2},\gamma_{3}^{2},\gamma_{4}^{2},\gamma_{1}^{3},\gamma_{3}^{3},\gamma_{5}^{3},\gamma_{3}^{4},\gamma_{4}^{4},\gamma_{5}^{4},\gamma_{1}^{5},\gamma_{6}^{5} \right]^{/}$$

$=\mathbf{S\gamma}$.

On the basis of the coefficient decomposition, the original regression model can be transformed into a new model, that is, $\mathbf{X}_{N\times p}\mathbf{\beta}_{p\times1}=\mathbf{X}_{N\times p}\mathbf{S}_{p\times u}\mathbf{\gamma}_{u\times1}=\mathop{\mathbf{X}}\limits^{\sim} _{N\times u}\mathbf{\gamma}_{u\times1}$. Equivalently, this new model can be constructed by duplicating the columns of overlapping variables in the original design matrix. For the new transformed model, the hazard function for subject $i$ in the Cox’s regression model is re-expressed as

.

**Appendix S.2: Simulation studies**

We consider survival data with a cohort size 300 as the training set, where each subject’s survival time follows the Cox’s proportional hazards model

$\lambda_{0}\left(t|\mathbf{e},\mathbf{x},\mathbf{w}\right)=10\exp\left(\mathbf{e}^{/}\mathbf{\alpha}+\mathbf{x}^{/}\mathbf{\beta}+\mathbf{w}^{/}\mathbf{\eta}\right)$,

with the covariates $\mathbf{e}$ and $\mathbf{x}$ jointly following a multivariate standard normal distribution with correlation $corr\left(e_{\cdot j},e_{\cdot k}\right)=0.3^{\left|j-k\right|}$ and $corr\left(x_{\cdot j},x_{\cdot k}\right)=0.5^{\left|j-k\right|}$, respectively. The censoring time distribution follows a uniform distribution. We then generate survival data, independent of the training data, with a cohort of size 100 as the test data to assess the prediction accuracy for different methods.

**S.2.1: Simulation Setting 1: equal group size data**

In this simulation study, we design 5 environmental variables and assume that the first 4 factors are related to the survival outcome, and the corresponding effects are 1.5, 2.25, 3, -1.5. The design matrix consists of 25 groups with each group having equal group sizes. The group size (number of genes in each group) and the overlapping structure (number of genes shared by two overlapping groups) are shown in Table S.1.

**Table S.1** Data structure for equal group size data

| Group | 1 | 2 | 3 | 4 | 5 | 6 | 7 | 8 | 9 | 10 | 11 | 12 | 13 | 14 | 15 | 16 | 17 | 18 | 19 | 20 | 21 | 22 | 23 | 24 | 25 |
| --- | --- | --- | --- | --- | --- | --- | --- | --- | --- | --- | --- | --- | --- | --- | --- | --- | --- | --- | --- | --- | --- | --- | --- | --- | --- |
| Gene Size | 23 | 23 | 23 | 23 | 23 | 23 | 23 | 23 | 23 | 23 | 23 | 23 | 23 | 23 | 23 | 23 | 23 | 23 | 23 | 23 | 23 | 23 | 23 | 23 | 23 |
| Overlapping | 3 3 0 3 3 0 3 3 0 3 3 0 0 3 0 0 3 0 3 3 0 0 3 0 | | | | | | | | | | | | | | | | | | | | | | | | |


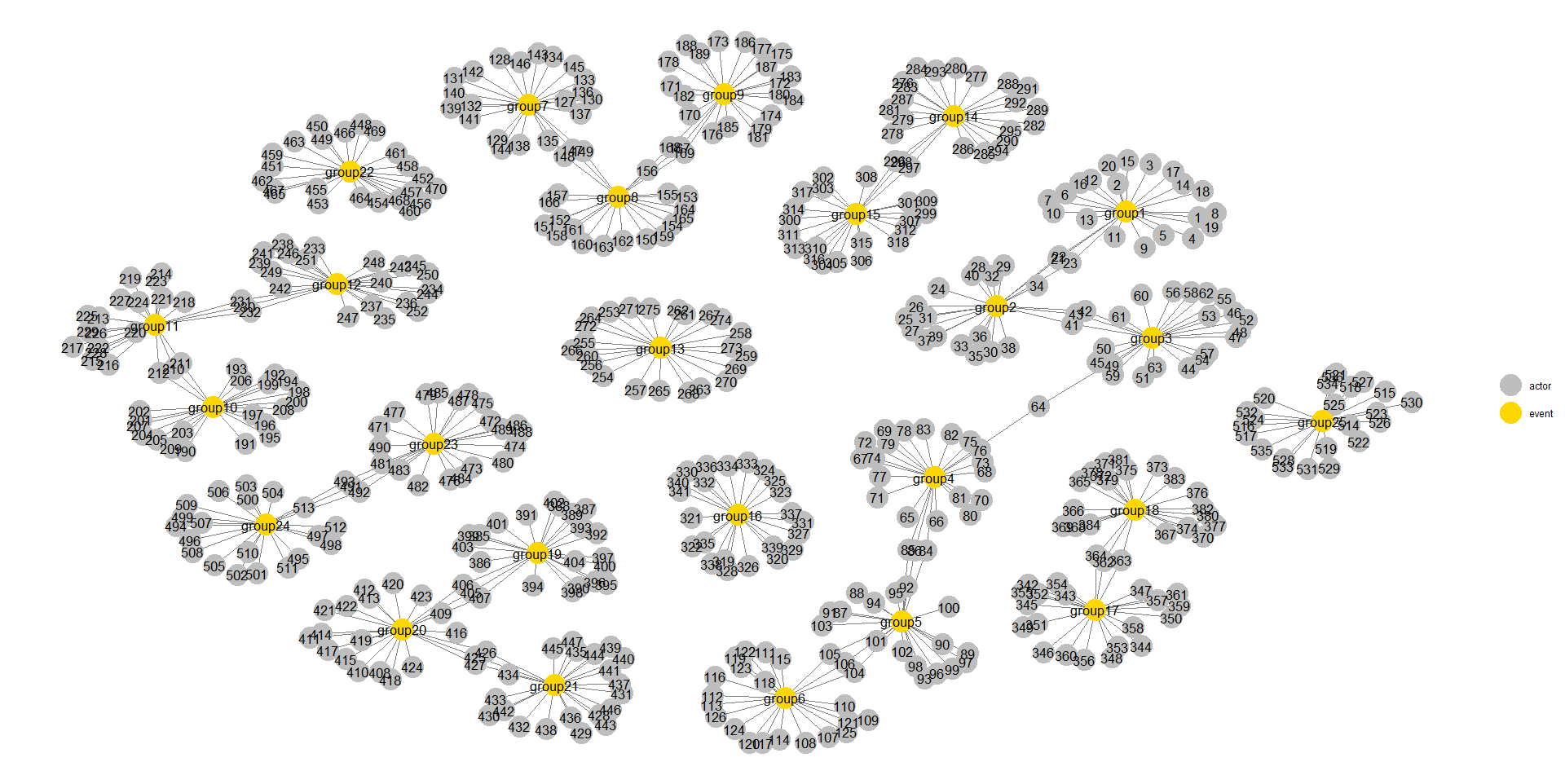


**Fig. S.1** gene network structure for equal group size data

For example, group 1 contains 23 genes, as group 2 does, and the two groups contain 43 unique genes, and 3 genes are shared by the two groups. As a result, there are 536 genes and 575 latent effects in this example. Fig. S.1 shows the gene indices of the groups in this data. Groups 1, 7, 13, and 19 are effective, and genes in each of them have constant latent effects of 3, 3, 2, and -2, respectively. Also, effective interactions (E1*G130, E1*G135, E2*G140) with the corresponding effects $(1.5,1.5,2)$ and (E2*G255, E3*G260, E3*G265) with the corresponding effects $(-1, -1.5, -2)$ in group 7 and group 13, respectively. The number of effective environments, genes and G-E interaction pairs is 102 among the total 3,221 environments, genes and G-E interaction pairs. We examine performances of different methods under a censoring rate of 30%, 50%, and 70%.

**S.2.2: Simulation Setting 2: small group size data**

In this simulation study, we design 5 environmental variables and assume that the first 4 factors are related to the survival outcome, and the corresponding effects are 1.5, 2.25, 3, -1.5. The design matrix consists of 5 groups with each group having different group sizes. The group size (number of genes in each group) and the overlapping structure (number of genes shared by two overlapping groups) are shown in Table S.2.

**Table S.2** Data structure for small group size data

| Group | 1 | 2 | 3 | 4 | 5 |
| --- | --- | --- | --- | --- | --- |
| Gene Size | 7 | 14 | 21 | 28 | 35 |
| Overlapping | 3 5 7 9 | | | | |


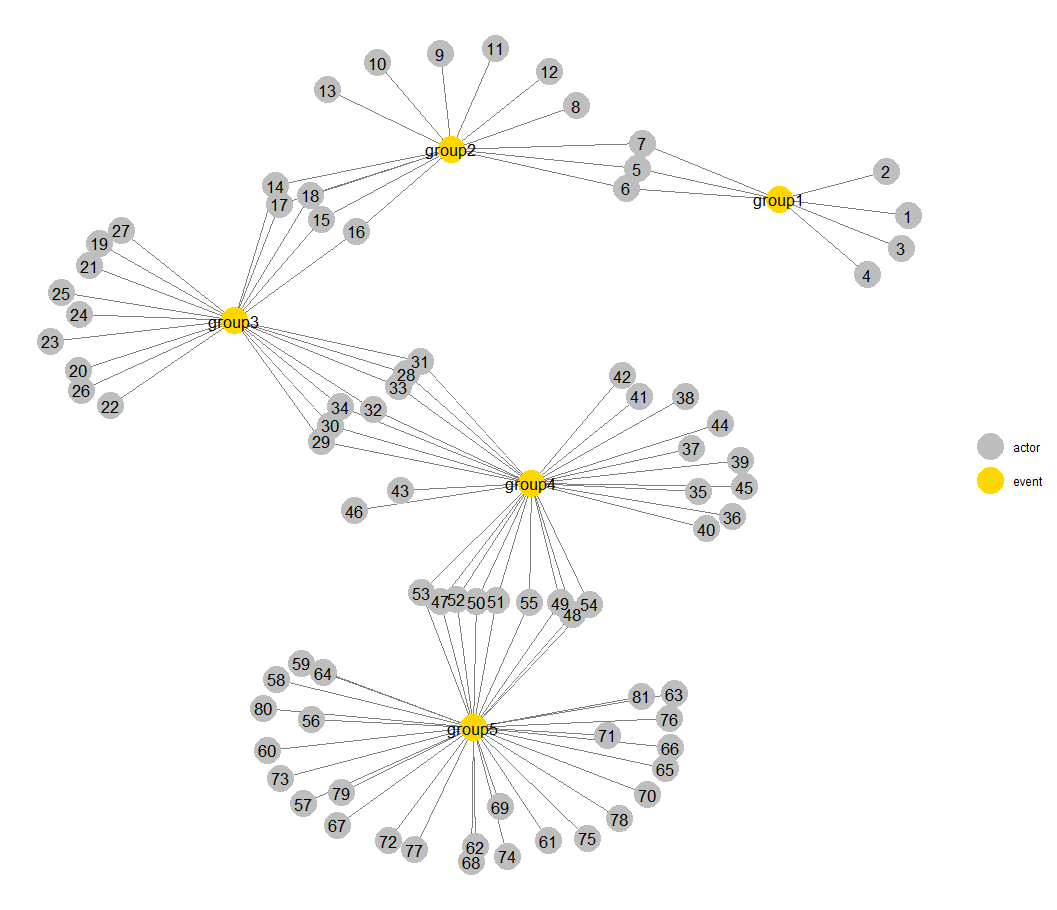


**Fig. S.2** gene network structure for small group size data

For example, group 1 and group 2 contains 7 and 14 genes, respectively. The two groups contain 18 unique genes, and 3 genes are shared by the two groups. As a result, there are 81 genes and 105 latent effects in this example. Fig. S.2 shows the gene indices of the groups in this data. Groups 2 and 4 are effective, and genes in each of them have constant latent effects of 3 and -2, respectively. Also, effective interactions (E1*G9, E1*G11, E2*G13) the corresponding effects $(1.5,1.5,2)$ and (E2*G36, E3*G39, E3*G42) with the corresponding effects $(-1, -1.5, -2)$ in group 2 and group 4, respectively. The number of effective environments, genes and G-E interaction pairs is 52 among the total 491 environments, genes and G-E interaction pairs. We examine performances of different methods under a censoring rate of 30%, 50%, and 70%.

From the simulation results shown in Tables 1 and S.3, the gene structure is complex, the OGS method using the Lasso or Ridge penalty performs substantially better than the “SIS Lasso”, “Ordinary Lasso”, “GSIS SCAD” methods in variable selection, estimation, and survival prediction. It can be seen from Table S.4 that when the gene structure is simple and the censoring rate is 30%, the performance of OGS with Lasso or Ridge penalty is worse than that of the “Ordinary Lasso” method, while when the censoring rate is higher (50% or 70%), the OGS with the Lasso or Ridge penalty performs better than the “Ordinary Lasso” in variable selection, estimation, and survival prediction.

**S.2.3: Simulation Setting 3: small group size data with some genes shared by three groups**

In this simulation study, the simulation setup was the same as the small group size data above, except that we designed a situation where some genes (14,15) were shared by three groups instead of just two. Figure S.3 shows the gene index for each group in this data. We still obtain similar numeric results patterns; these corresponding results are shown in Table S.8.

**Fig. S.3** gene network structure for small group size data with some genes shared by three groups

**Table S.3**

The median of the measures out of 200 simulation replications for different approaches with equal group size data

|  | Oracle | | GSIS  SCAD | | SIS  Lasso | Ordinary  Lasso | OGS  Ridge | OGS  Lasso |
| --- | --- | --- | --- | --- | --- | --- | --- | --- |
|  | | Censoring rate = 30% | | | | | | |
| RMSE | 0.4253 | | 0.3393 | | 0.4337 | 0.4304 | 0.4371 | 0.4035 |
| P.int | 1.0000 | | 0.0000 | | 0.0000 | 0.0000 | 0.5000 | 0.3333 |
| Sen. | 1.0000 | | 0.9020 | | 0.3235 | 0.6569 | 0.9706 | 0.9412 |
| Spe. | 1.0000 | | 1.0000 | | 0.9962 | 0.9843 | 0.9269 | 0.9894 |
| C.model | 102.0000 | | 92.0000 | | 45.0000 | 116.0000 | 327.0000 | 130.0000 |
| Deviance | -105.6688 | | | -108.0987 | -55.3600 | -98.3199 | -63.9476 | -220.9675 |
| C-index | 0.8495 | | 0.9362 | | 0.7765 | 0.8526 | 0.8804 | 0.9406 |
| AUC | 0.9196 | | 0.9780 | | 0.8488 | 0.9278 | 0.9478 | 0.9860 |
|  | | Censoring rate = 50% | | | | | | |
| RMSE | 0.4160 | | 0.4403 | | 0.4337 | 0.4332 | 0.4371 | 0.4300 |
| P.int | 1.0000 | | 0.0000 | | 0.0000 | 0.0000 | 0.5000 | 0.0000 |
| Sen. | 1.0000 | | 0.6765 | | 0.3039 | 0.5294 | 0.7451 | 0.5392 |
| Spe. | 1.0000 | | 1.0000 | | 0.9955 | 0.9849 | 0.9269 | 0.9945 |
| C.model | 102.0000 | | 69.0000 | | 45.0000 | 100.0000 | 281.0000 | 72.0000 |
| Deviance | -75.6567 | | -21.0076 | | -42.9492 | -61.9634 | -47.6737 | -68.6489 |
| C-index | 0.8675 | | 0.8122 | | 0.7847 | 0.8307 | 0.8466 | 0.8386 |
| AUC | 0.9225 | | 0.8647 | | 0.8390 | 0.8894 | 0.9010 | 0.9253 |
|  | | Censoring rate = 70% | | | | | | |
| RMSE | 0.4079 | | 0.4415 | | 0.4349 | 0.4358 | 0.4365 | 0.4335 |
| P.int | 1.0000 | | 0.0000 | | 0.0000 | 0.0000 | 0.5000 | 0.0000 |
| Sen. | 1.0000 | | 0.4510 | | 0.2549 | 0.3824 | 0.5196 | 0.3431 |
| Spe. | 1.0000 | | 1.0000 | | 0.9949 | 0.9872 | 0.9628 | 0.9958 |
| C.model | 102.0000 | | 46.0000 | | 42.0000 | 79.0000 | 189.0000 | 49.0000 |
| Deviance | 120.5683 | | -7.3415 | | -24.3494 | -31.1085 | -29.6446 | -35.6544 |
| C-index | 0.8317 | | 0.8025 | | 0.7851 | 0.8152 | 0.8385 | 0.8250 |
| AUC | 0.8618 | | 0.8315 | | 0.8193 | 0.8446 | 0.8743 | 0.8587 |

**Table S.4**

The median of the measures out of 200 simulation replications for different approaches with small group size data

|  | Oracle | | GSIS  SCAD | | SIS  Lasso | Ordinary  Lasso | OGS  Ridge | OGS  Lasso |
| --- | --- | --- | --- | --- | --- | --- | --- | --- |
|  | | Censoring rate = 30% | | | | | | |
| RMSE | 0.6937 | | 0.6281 | | 0.6721 | 0.5123 | 0.6301 | 0.5842 |
| P.int | 1.0000 | | 0.0000 | | 0.0000 | 1.0000 | 0.5000 | 0.5000 |
| Sen. | 1.0000 | | 0.8077 | | 0.7500 | 1.0000 | 0.9423 | 0.9423 |
| Spe. | 1.0000 | | 1.0000 | | 0.9841 | 0.8223 | 0.6856 | 0.9226 |
| C.model | 52.0000 | | 42.0000 | | 46.0000 | 130.0000 | 187.0000 | 83.0000 |
| Deviance | -151.2621 | | | -166.7692 | -169.3500 | -333.7422 | -231.0364 | -276.6498 |
| C-index | 0.9122 | | 0.9207 | | 0.9116 | 0.9792 | 0.9480 | 0.9638 |
| AUC | 0.9690 | | 0.9764 | | 0.9708 | 0.9976 | 0.9868 | 0.9948 |
|  | | Censoring rate = 50% | | | | | | |
| RMSE | 0.6774 | | 0.6293 | | 0.6772 | 0.6087 | 0.7298 | 0.5776 |
| P.int | 1.0000 | | 0.0000 | | 0.0000 | 1.0000 | 0.5000 | 0.5000 |
| Sen. | 1.0000 | | 0.8077 | | 0.7308 | 1.0000 | 0.9423 | 0.9423 |
| Spe. | 1.0000 | | 1.0000 | | 0.9818 | 0.8884 | 0.5330 | 0.9134 |
| C.model | 52.0000 | | 42.0000 | | 45.0000 | 101.0000 | 257.0000 | 87.0000 |
| Deviance | -131.1451 | | | -127.5112 | -129.0066 | -207.5030 | -56.7074 | -215.1998 |
| C-index | 0.9238 | | 0.9257 | | 0.9197 | 0.9695 | 0.9265 | 0.9706 |
| AUC | 0.9676 | | 0.9722 | | 0.9651 | 0.9937 | 0.9708 | 0.9940 |
|  | | Censoring rate = 70% | | | | | | |
| RMSE | 0.6853 | | 0.7092 | | 0.6868 | 0.6776 | 0.6937 | 0.6541 |
| P.int | 1.0000 | | 0.0000 | | 0.1667 | 0.5000 | 0.5000 | 0.5000 |
| Sen. | 1.0000 | | 0.8077 | | 0.6538 | 0.8462 | 0.9423 | 0.9038 |
| Spe. | 1.0000 | | 1.0000 | | 0.9772 | 0.9248 | 0.6856 | 0.9499 |
| C.model | 52.0000 | | 42.0000 | | 44.0000 | 78.0000 | 187.0000 | 69.0000 |
| Deviance | -72.8806 | | -44.4240 | | -76.1749 | -92.4807 | -74.0674 | -110.7252 |
| C-index | 0.9113 | | 0.8972 | | 0.9198 | 0.9431 | 0.9327 | 0.9567 |
| AUC | 0.9437 | | 0.9289 | | 0.9550 | 0.9700 | 0.9665 | 0.9806 |

**Table S.5**

The average of the measures out of 200 simulation replications for different approaches with varied group size data based on a small cohort size 150

|  | Oracle | | GSIS  SCAD | SIS  Lasso | Ordinary  Lasso | OGS  Ridge | OGS  Lasso |
| --- | --- | --- | --- | --- | --- | --- | --- |
|  | | Censoring rate = 30% | | | | | |
| RMSE | 0.3431 | | 0.3673 | 0.3689 | 0.3706 | 0.3681 | 0.3620 |
| P.int | 1.0000 | | 0.0000 | 0.0000 | 0.0000 | 0.5000 | 0.0000 |
| Sen. | 1.0000 | | 0.8901 | 0.1429 | 0.1868 | 0.9670 | 0.5220 |
| Spe. | 1.0000 | | 0.9979 | 0.9959 | 0.9949 | 0.9310 | 0.9962 |
| C.model | 91.0000 | | 84.5000 | 25.0000 | 33.0000 | 289.0000 | 59.0000 |
| Deviance | 75.5584 | | -16.8927 | 0.6930 | -3.7731 | -21.6700 | -33.0625 |
| C-index | 0.7892 | | 0.7788 | 0.6583 | 0.6780 | 0.8452 | 0.8124 |
| AUC | 0.8640 | | 0.8496 | 0.7032 | 0.7280 | 0.9160 | 0.8864 |
|  | | Censoring rate = 50% | | | | | |
| RMSE | 11.7967 | | 0.3684 | 0.3689 | 0.3703 | 0.3684 | 0.3656 |
| P.int | 1.0000 | | 0.0000 | 0.0000 | 0.0000 | 0.5000 | 0.0000 |
| Sen. | 1.0000 | | 0.8901 | 0.1319 | 0.1868 | 0.9451 | 0.3846 |
| Spe. | 1.0000 | | 0.9990 | 0.9957 | 0.9935 | 0.9173 | 0.9966 |
| C.model | 91.0000 | | 81.0000 | 25.0000 | 37.0000 | 323.0000 | 48.0000 |
| Deviance | 2019.7945 | | -5.7707 | 1.4012 | -2.6864 | -14.0520 | -17.2679 |
| C-index | 0.7118 | | 0.7366 | 0.6667 | 0.6882 | 0.8139 | 0.7819 |
| AUC | 0.7521 | | 0.7741 | 0.6951 | 0.7214 | 0.8695 | 0.8306 |
|  | | Censoring rate = 70% | | | | | |
| RMSE | 0.4867 | | 0.3698 | 0.3696 | 0.3709 | 0.3695 | 0.3682 |
| P.int | 1.0000 | | 0.0000 | 0.0000 | 0.0000 | 0.5000 | 0.0000 |
| Sen. | 1.0000 | | 0.5275 | 0.0989 | 0.1429 | 0.5714 | 0.2033 |
| Spe. | 1.0000 | | 0.9993 | 0.9955 | 0.9938 | 0.9667 | 0.9973 |
| C.model | 91.0000 | | 54.0000 | 23.0000 | 30.0000 | 149.0000 | 28.0000 |
| Deviance | 686.3433 | | 2.2505 | 4.7627 | 0.4380 | -3.6992 | -2.1802 |
| C-index | 0.7504 | | 0.6936 | 0.6487 | 0.6664 | 0.7568 | 0.7245 |
| AUC | 0.7746 | | 0.7140 | 0.6743 | 0.6819 | 0.7834 | 0.7387 |

**Table S.6**

The median of the measures out of 200 simulation replications for different approaches with equal group size data based on a small cohort size 150

|  | Oracle | | GSIS  SCAD | SIS  Lasso | Ordinary  Lasso | OGS  Ridge | OGS  Lasso |
| --- | --- | --- | --- | --- | --- | --- | --- |
|  | | Censoring rate = 30% | | | | | |
| RMSE | 0.4181 | | 0.4395 | 0.4392 | 0.4409 | 0.4385 | 0.4342 |
| P.int | 1.0000 | | 0.0000 | 0.0000 | 0.0000 | 0.5000 | 0.0000 |
| Sen. | 1.0000 | | 0.9020 | 0.1176 | 0.1569 | 0.9412 | 0.3971 |
| Spe. | 1.0000 | | 1.0000 | 0.9962 | 0.9952 | 0.9269 | 0.9968 |
| C.model | 102.0000 | | 92.0000 | 25.0000 | 32.0000 | 304.0000 | 53.0000 |
| Deviance | 205.8245 | | -14.5907 | -2.2799 | -3.8834 | -20.0813 | -27.4677 |
| C-index | 0.7358 | | 0.7905 | 0.6697 | 0.6798 | 0.8253 | 0.7874 |
| AUC | 0.8024 | | 0.8584 | 0.7160 | 0.7336 | 0.9008 | 0.8720 |
|  | | Censoring rate = 50% | | | | | |
| RMSE | 7.5866 | | 0.4408 | 0.4392 | 0.4408 | 0.4389 | 0.4362 |
| P.int | 1.0000 | | 0.0000 | 0.0000 | 0.0000 | 0.5000 | 0.0000 |
| Sen. | 1.0000 | | 0.6765 | 0.1176 | 0.1520 | 0.7451 | 0.2941 |
| Spe. | 1.0000 | | 1.0000 | 0.9962 | 0.9945 | 0.9554 | 0.9971 |
| C.model | 102.0000 | | 69.0000 | 25.0000 | 32.0000 | 235.0000 | 40.0000 |
| Deviance | 15948.2850 | | -5.8180 | -0.0996 | -2.8429 | -13.4026 | -14.8090 |
| C-index | 0.7069 | | 0.7734 | 0.6700 | 0.6844 | 0.8057 | 0.7665 |
| AUC | 0.7467 | | 0.8243 | 0.7080 | 0.7266 | 0.8648 | 0.8200 |
|  | | Censoring rate = 70% | | | | | |
| RMSE | 0.5192 | | 0.4420 | 0.4399 | 0.4412 | 0.4398 | 0.4384 |
| P.int | 1.0000 | | 0.0000 | 0.0000 | 0.0000 | 0.5000 | 0.0000 |
| Sen. | 1.0000 | | 0.4510 | 0.0833 | 0.1078 | 0.5196 | 0.1863 |
| Spe. | 1.0000 | | 1.0000 | 0.9955 | 0.9945 | 0.9628 | 0.9978 |
| C.model | 102.0000 | | 46.0000 | 22.0000 | 30.0000 | 166.0000 | 27.0000 |
| Deviance | 664.5979 | | 0.8024 | 5.9987 | 1.7362 | -5.2587 | -3.2394 |
| C-index | 0.7273 | | 0.7388 | 0.6352 | 0.6532 | 0.7786 | 0.7369 |
| AUC | 0.7620 | | 0.7653 | 0.6406 | 0.6789 | 0.8020 | 0.7534 |

**Table S.7**

The median of the measures out of 200 simulation replications for different approaches with small group size data based on a small cohort size 150

|  | Oracle | | GSIS  SCAD | SIS  Lasso | Ordinary  Lasso | OGS  Ridge | OGS  Lasso |
| --- | --- | --- | --- | --- | --- | --- | --- |
|  | | Censoring rate = 30% | | | | | |
| RMSE | 0.6828 | | 0.6892 | 0.7127 | 0.6931 | 0.7324 | 0.6576 |
| P.int | 1.0000 | | 0.0000 | 0.0000 | 0.3333 | 0.5000 | 0.3333 |
| Sen. | 1.0000 | | 0.8077 | 0.4135 | 0.7885 | 0.9423 | 0.8846 |
| Spe. | 1.0000 | | 1.0000 | 0.9909 | 0.9522 | 0.6856 | 0.9658 |
| C.model | 52.0000 | | 42.0000 | 26.0000 | 62.0000 | 187.0000 | 61.0000 |
| Deviance | -57.4652 | | -34.2341 | -35.6424 | -66.5847 | -24.9655 | -90.6250 |
| C-index | 0.8745 | | 0.8826 | 0.8131 | 0.8894 | 0.8872 | 0.9280 |
| AUC | 0.9392 | | 0.9456 | 0.8856 | 0.9552 | 0.9584 | 0.9776 |
|  | | Censoring rate = 50% | | | | | |
| RMSE | 0.6555 | | 0.7196 | 0.7129 | 0.7068 | 0.7326 | 0.6866 |
| P.int | 1.0000 | | 0.0000 | 0.0000 | 0.1667 | 0.5000 | 0.1667 |
| Sen. | 1.0000 | | 0.8077 | 0.3846 | 0.6538 | 0.9423 | 0.7692 |
| Spe. | 1.0000 | | 1.0000 | 0.9886 | 0.9544 | 0.6856 | 0.9704 |
| C.model | 52.0000 | | 42.0000 | 25.0000 | 54.0000 | 187.0000 | 52.0000 |
| Deviance | -32.6912 | | -13.5442 | -26.1059 | -39.0841 | -18.1482 | -51.4409 |
| C-index | 0.8816 | | 0.8245 | 0.8215 | 0.8742 | 0.8982 | 0.9067 |
| AUC | 0.9356 | | 0.8848 | 0.8801 | 0.9303 | 0.9473 | 0.9520 |
|  | | Censoring rate = 70% | | | | | |
| RMSE | 0.6773 | | 0.7167 | 0.7168 | 0.7178 | 0.7326 | 0.7095 |
| P.int | 1.0000 | | 0.0000 | 0.0000 | 0.1667 | 0.5000 | 0.1667 |
| Sen. | 1.0000 | | 0.8077 | 0.3269 | 0.4615 | 0.9423 | 0.5577 |
| Spe. | 1.0000 | | 1.0000 | 0.9841 | 0.9613 | 0.7904 | 0.9795 |
| C.model | 52.0000 | | 42.0000 | 23.0000 | 43.0000 | 117.0000 | 38.0000 |
| Deviance | 77.5074 | | -0.9303 | -12.6817 | -17.5005 | -11.4831 | -19.4573 |
| C-index | 0.8257 | | 0.8112 | 0.8235 | 0.8533 | 0.8718 | 0.8674 |
| AUC | 0.8525 | | 0.8499 | 0.8578 | 0.8911 | 0.9081 | 0.9053 |

**Table S.8**

The median of the measures out of 200 simulation replications for different approaches with small group size data and some genes shared by three groups based on a small cohort size 300

|  | Oracle | | GSIS  SCAD | | SIS  Lasso | Ordinary  Lasso | OGS  Ridge | OGS  Lasso |
| --- | --- | --- | --- | --- | --- | --- | --- | --- |
|  | | Censoring rate = 30% | | | | | | |
| RMSE | 0.6939 | | 0.6275 | | 0.6737 | 0.5143 | 0.6408 | 0.5826 |
| P.int | 1.0000 | | 0.0000 | | 0.0000 | 1.0000 | 0.5000 | 0.5000 |
| Sen. | 1.0000 | | 0.8077 | | 0.7308 | 1.0000 | 0.9423 | 0.9423 |
| Spe. | 1.0000 | | 1.0000 | | 0.9841 | 0.8246 | 0.6856 | 0.9169 |
| C.model | 52.0000 | | 42.0000 | | 46.0000 | 129.0000 | 187.0000 | 85.0000 |
| Deviance | -151.1481 | | | -166.6798 | -166.1883 | -330.5469 | -223.8281 | -280.4980 |
| C-index | 0.9122 | | 0.9198 | | 0.9105 | 0.9788 | 0.9438 | 0.9649 |
| AUC | 0.9694 | | 0.9760 | | 0.9710 | 0.9980 | 0.9856 | 0.9952 |
|  | | Censoring rate = 50% | | | | | | |
| RMSE | 0.6783 | | 0.6267 | | 0.6718 | 0.6111 | 0.6610 | 0.5852 |
| P.int | 1.0000 | | 0.0000 | | 0.0000 | 1.0000 | 0.5000 | 0.5000 |
| Sen. | 1.0000 | | 0.8077 | | 0.7308 | 1.0000 | 0.9423 | 0.9423 |
| Spe. | 1.0000 | | 1.0000 | | 0.9829 | 0.8884 | 0.6856 | 0.9248 |
| C.model | 52.0000 | | 42.0000 | | 46.0000 | 101.0000 | 187.0000 | 82.0000 |
| Deviance | -130.7381 | | | -121.1882 | -132.0395 | -206.5147 | -144.8922 | -210.4170 |
| C-index | 0.9245 | | 0.9254 | | 0.9222 | 0.9696 | 0.9377 | 0.9683 |
| AUC | 0.9688 | | 0.9734 | | 0.9683 | 0.9941 | 0.9751 | 0.9944 |
|  | | Censoring rate = 70% | | | | | | |
| RMSE | 0.6857 | | 0.6957 | | 0.6853 | 0.6787 | 0.6864 | 0.6493 |
| P.int | 1.0000 | | 0.0000 | | 0.1667 | 0.6667 | 0.5000 | 0.5000 |
| Sen. | 1.0000 | | 0.8077 | | 0.6538 | 0.8462 | 0.9423 | 0.9038 |
| Spe. | 1.0000 | | 1.0000 | | 0.9784 | 0.9248 | 0.6856 | 0.9522 |
| C.model | 52.0000 | | 42.0000 | | 44.0000 | 77.0000 | 187.0000 | 69.0000 |
| Deviance | -71.8149 | | -42.1147 | | -76.0652 | -92.9992 | -70.9941 | -109.5113 |
| C-index | 0.9117 | | 0.9018 | | 0.9200 | 0.9415 | 0.9429 | 0.9588 |
| AUC | 0.9450 | | 0.9358 | | 0.9538 | 0.9715 | 0.9725 | 0.9821 |

**Appendix S.3: Real data application**

**S.3.1 Real data application: TCGA HNSCC data**

The TCGA HNSCC RNA-Seq expression data, together with the phenotype data containing the survival time and censoring status data, can be downloaded from the R package ’TCGAbiolinks’, or ’UCSCXenaTools’. After excluding patients with missing survival time data, our analysis is focused on the subset of the TCGA HNSCC data with 517 patients and 20,501 gene expression variables. The censoring rate of the survival time in the data is about 58%. The TCGA HNSCC clinical information data can be obtained from the ’FireBrowse’ database.

Due to the number of cancer-related genes is expected to be limited, we conduct prescreening using non-parametric IPCW Kendall’s tau correlation, which can also improve stability for feature selection. The top 2,000 genes with the largest absolute IPCW Kendall’s tau correlation are selected for downstream analysis. The five E factors analyzed including AJCC pathologic stage nodes, AJCC pathologic stage tumor, age, gender, and ICD O3 site. Summary information for these clinical variables is reported in the Table 3. Some of the clinical variables contain missing values, and we use the sparse boosting method in the R package "*GEInter*" to perform multiple imputation for the missing values in the clinical variables.

The different annotated gene sets databases including (GO cellular component, GO molecular function, KEEG) are used for the OGS approach, we then take 10 random splits of the whole data into 413:104 training/test sets of data to evaluate the performance of all methods for survival prediction in the TCGA HNSCC data. The results are shown in Table S.9 From these results, the OGS approach still performs better than the other methods.

When applying the OGS with the Lasso penalty to the entire data based on the GO biological process database, we identified several major and interacting biomarkers and estimated the corresponding parameters, as shown in Table S.10. We find the clinical variable "Age" interacts with several genes, and most of these genes, such as “*CAMP*”, “*DEFB1*”, “*MAP2K7*” have been shown to be related to HNSCC. And “Age” factor has been shown to be related to HNSCC.

**Table S.9**

Results (median of prediction accuracy of different methods with different annotated gene sets databases in the TCGA HNSCC data over 10 random splits of 413:104 training /test sets)

|  | GSIS  SCAD | SIS  Lasso | Ordinary  Lasso | OGS  Ridge | OGS  Lasso | PTReg |
| --- | --- | --- | --- | --- | --- | --- |
| GO cellular component | | | | | | |
| Cox-test | 0.3334 | 0.0729 | 0.0259 | 0.0020 | 0.0055 | 0.1100 |
| LR-test | 0.4058 | 0.1257 | 0.0694 | 0.0094 | 0.0066 | 0.1672 |
| Deviance | 31.0294 | 7.8314 | 4.7617 | -1.2845 | 6.9222 | 44.6680 |
| C-index | 0.5612 | 0.6201 | 0.6415 | 0.6803 | 0.6477 | 0.5970 |
| AUC | 0.5645 | 0.6324 | 0.6497 | 0.6955 | 0.6608 | 0.6169 |
| GO molecular function | | | | | | |
| Cox-test | 0.2844 | 0.0059 | 0.0019 | 0.0004 | 0.0023 | 0.0521 |
| LR-test | 0.3989 | 0.0764 | 0.0416 | 0.0068 | 0.0383 | 0.1035 |
| Deviance | 56.7259 | 6.3314 | 3.3692 | -0.7468 | 6.4043 | 41.2061 |
| C-index | 0.5512 | 0.6288 | 0.6368 | 0.6652 | 0.6378 | 0.6003 |
| AUC | 0.4325 | 0.6359 | 0.6509 | 0.6638 | 0.6250 | 0.6090 |
| KEEG | | | | | | |
| Cox-test | 0.3332 | 0.0146 | 0.0022 | 0.0002 | 0.0048 | 0.0077 |
| LR-test | 0.6420 | 0.0815 | 0.0264 | 0.0054 | 0.0316 | 0.0824 |
| Deviance | 26.6164 | 9.3717 | 6.8386 | 1.1858 | 9.3621 | 48.0979 |
| C-index | 0.5449 | 0.6239 | 0.6361 | 0.6779 | 0.6344 | 0.6113 |
| AUC | 0.4655 | 0.6669 | 0.6566 | 0.7087 | 0.6562 | 0.6194 |

**Table S.10**

Analysis of the TCGA HNSCC data using the OGS approach: coefficients of identified interactions

|  | AJCC pathologic  nodes | Age |
| --- | --- | --- |
| "ADCY2" "AIG1" "CAMP" "CELSR3" "COBL" "CRTAC1" "DEFB1" "EDA"  "EFNB2" "EPHA3" "EPHX3" "EZR" "FAT4" "GRIA3" "GRIN3A" "HMMR"  "ITGA4" "LIF" "MAP2K7" "MNX1" "MOGAT2" "NLGN4Y" "OTUD7A" "PAX8"  "PGK1" "PTX3" "RFPL1" "RYR2" "S1PR4" "SEMA3A" "SFRP1" "SLC26A1"  "SPINK1" "SPINK6" "SYT1" "SYT2" "THBS1" "TIMP4" "TPP1" "TRIML2"  "TTBK1" "VSIG4" | 0.00253 | 0.00097  0.00089  -0.00057  -0.00141  0.00106  0.00088  -0.00102  0.00214  0.00031  -0.00125  -0.00004  0.00200  -0.00147  -0.00103  -0.00044  0.00051  -0.00160  0.00102  -0.00156  0.00048  -0.00023  -0.00043  -0.00246  -0.00194  0.00097  0.00030  -0.00087  0.00031  -0.00015  0.00063  -0.00027  -0.00044  0.00056  -0.00187  0.00041  -0.00175  0.00064  0.00082  0.00029  0.00173  -0.00174  0.00144 |

**S.4.2 Real data application: TCGA ESCA data**

The TCGA ESCA RNA-Seq expression data, together with the phenotype data containing the survival time and censoring status data can be downloaded from the R package ‘TCGAbiolinks’, or ‘UCSCXenaTools’. After excluding patients with missing survival time data, our analysis is focused on the subset of the TCGA ESCA data with 368 patients and 20,501 gene expression variables. The censoring rate in the data is about 58%. The TCGA ESCA clinical information data can be obtained from the ‘FireBrowse’ database.

Due to the number of cancer-related genes is expected to be limited, we conduct prescreening using non-parametric IPCW Kendall’s tau correlation, which can also improve stability for feature selection. The top 2,000 genes with the largest absolute IPCW Kendall’s tau correlation are selected for downstream analysis. The seven E effects analyzed including age, gender, esophageal tumor central location, person neoplasm cancer status, rice, BMI, and AJCC pathologic stage. The corresponding clinical variables information is reported in the Table 5. However, several clinical variables we considered are missingness, so the multiple imputation approach based on sparse boosting method is utilized to accommodate missingness in clinical measurements. The multiple imputation approach can be performed by “GEInter” package friendly.

The different annotated gene sets databases including (GO-CC, GO-MF, KEEG) are also used for the OGS approach, we also take ten random splits of the whole data into 294:74 training/test sets of data to evaluate the performance of all methods for survival prediction in the TCGA ESCA data. The results are shown in Table S.11. From these results, the OGS approach still performs better than the other methods.

When applying the OGS with Lasso penalty for whole data based on the GO biological process database, we identify several main and interacting biomarkers and estimate the correspondence parameters, which are shown in Table S.12. We find the clinical variable "Age" interacts with several genes, and most of these genes, such as “*CD40LG*”, “*DEK*”, “*IL6*” have been shown to be related to ESCA. And two “Weight” and “Age” factors have been shown to be related to HNSCC.

**Table S.11**

Results (median of prediction accuracy of different methods with different annotated gene sets databases in the TCGA ESCA data over 10 random splits of 294:74 training /test sets)

|  | GSIS  SCAD | SIS  Lasso | Ordinary  Lasso | OGS  Ridge | OGS  Lasso | PTReg |
| --- | --- | --- | --- | --- | --- | --- |
| GO cellular component | | | | | | |
| Cox-test | 0.4349 | 0.0007 | 5.05e-10 | 2.31e-10 | 4.17e-09 | 0.3362 |
| LR-test | 0.5668 | 0.0138 | 2.20e-08 | 1.08e-08 | 8.74e-07 | 0.3979 |
| Deviance | 104.3625 | 12.0324 | -35.9977 | -39.6518 | -38.4662 | 9.0938 |
| C-index | 0.5423 | 0.6162 | 0.8717 | 0.8967 | 0.8856 | 0.6157 |
| AUC | 0.5208 | 0.5779 | 0.8755 | 0.8993 | 0.8806 | 0.6859 |
| GO molecular function | | | | | | |
| Cox-test | 0.2982 | 0.0414 | 4.41e-07 | 2.07e-09 | 0.0017 | 0.3890 |
| LR-test | 0.3679 | 0.1142 | 5.68e-05 | 9.79e-07 | 0.0308 | 0.4155 |
| Deviance | 130.2006 | 9.6961 | -32.5743 | -33.4680 | -36.6475 | 37.1800 |
| C-index | 0.5834 | 0.6257 | 0.8437 | 0.8621 | 0.8464 | 0.6165 |
| AUC | 0.6039 | 0.6100 | 0.8718 | 0.8920 | 0.8823 | 0.6785 |
| KEEG | | | | | | |
| Cox-test | 0.4943 | 0.0003 | 1.77e-09 | 2.51e-10 | 2.76e-06 | 0.2635 |
| LR-test | 0.5370 | 0.0455 | 2.14e-08 | 1.24e-09 | 2.03e-05 | 0.2684 |
| Deviance | 481.6366 | 7.1601 | -16.5530 | -45.7797 | -4.5670 | 47.1708 |
| C-index | 0.5163 | 0.6599 | 0.8457 | 0.8905 | 0.8045 | 0.6501 |
| AUC | 0.4538 | 0.6518 | 0.8733 | 0.9240 | 0.8202 | 0.7630 |

**Table S.12**

Analysis of the TCGA ESCA data using the OGS approach: coefficients of identified interactions

|  | Weight | Age |
| --- | --- | --- |
| "ADD1"  "ANKFY1"  "AQP8"  "ATP2B3"  "B2M"  "BCAP31"  "C1QC"  "CARD11"  "CCR6"  "CCT4"  "CD40LG"  "CDH6"  "CDHR4"  "CDKN2A"  "CLDN1"  "CRABP1"  "DCHS2"  "DEK"  "DHX58"  "DLL1"  "DVL1"  "EFHB"  "ESM1"  "FDXACB1"  "FER1L5"  "FIS1"  "GALNS"  "HCN1"  "HES1"  "HES5"  "HMX2"  "HOXB3"  "HSPH1"  "IL17A"  "IL6"  "INCENP"  "KAT2A"  "KDM4D"  "MAML2"  "MDM2"  "MID1"  "MMP12"  "MRPL20"  "MRPL34"  "MT1G"  "MYPN"  "NINJ2"  "NLGN4Y"  "PARP3"  "PCDH11X"  "PCDHGB1"  "PUS10"  "RBM3"  "RETSAT"  "RUNX1"  "SLC1A4"  "SLC38A7"  "STC2"  "TAF9B"  "TEX19"  "TLR1"  "TPM1"  "TRIOBP"  "VPREB3"  "XCL1"  "YAP1"  "YIPF6"  "ZNF91" | 0.00256  0.00847  0.00269  0.00824 | -0.00307  -0.02662  0.00667  0.00553  0.00349  -0.00019  0.00348  0.00467  0.00930  0.01508  -0.00056  -0.00222  -0.00298  0.00997  0.00379  -0.00335  0.00732  -0.01064  0.00212  -0.00961  -0.00028  0.00228  -0.01398  -0.01161  0.00188  -0.00377  -0.01405  -0.00440  -0.00098  0.00045  0.00446  0.01508  -0.00475  -0.01212  -0.00840  0.00845  0.01568  0.00089  -0.00828  0.00871  -0.00283  0.00457  0.01483  0.00355  0.00427  0.00000  0.00600  0.00114  -0.00699  -0.00762  -0.02495  0.00002  0.00798  0.01013  0.00130  0.00745  -0.01209  0.01316  -0.00098  -0.00414  0.01157  -0.01127  0.01610  0.00367 |
